# Probing Steady‐State Carrier Properties and Charge Transport in Covalent Organic Framework by Frequency‐Domain Terahertz Spectroscopy

**DOI:** 10.1002/anie.3669544

**Published:** 2026-05-01

**Authors:** Satyapriya Nath, Saiprakash Rout, Mahalaxmi Samal, Snehal Haldankar, Md Habib Ahsan, Anol Mondal, Sudeep Tiwari, Adithyan Puthukkudi, Avani KV, Ashis K. Nandy, Shriganesh S. Prabhu, Himansu S. Biswal, Xinliang Feng, Bishnu P. Biswal

**Affiliations:** ^1^ School of Chemical Sciences National Institute of Science Education and Research (NISER) Bhubaneswar Khurda Odisha India; ^2^ Homi Bhabha National Institute (HBNI) Training School Complex Mumbai India; ^3^ Department of Condensed Matter Physics and Materials Science Tata Institute of Fundamental Research Mumbai Maharashtra India; ^4^ School of Physical Sciences National Institute of Science Education and Research (NISER) Bhubaneswar Khurda Odisha India; ^5^ Centre for Interdisciplinary Sciences National Institute of Science Education and Research (NISER) Bhubaneswar Khurda Odisha India; ^6^ Center For Advancing Electronics Dresden (cfaed) & Faculty of Chemistry and Food Chemistry Technische Universität Dresden Dresden Germany; ^7^ Max Planck Institute of Microstructure Physics Halle (Saale) Germany

**Keywords:** charge transport, covalent organic framework, frequency domain THz spectroscopy, steady‐state carrier properties, terahertz spectroscopy

## Abstract

Terahertz (THz) spectroscopy is an emerging tool for probing charge transport and optical properties in covalent organic frameworks (COFs). Existing studies have predominantly relied on time‐resolved THz spectroscopy (TRTS) to investigate photoexcited carriers, with only one report on time‐dependent THz spectroscopy (TDTS) to understand ground‐state carriers within a narrow spectral window. Frequency‐domain THz spectroscopy (FDTS), which offers high spectral resolution across the far–infrared‐THz range remains unexplored. Herein, we employ FDTS to investigate steady‐state carrier transport in a COF (TTC‐PD) and amorphous frameworks (TTC‐DTO and TTC‐PD (amor)), along with their molecular analogues. Frequency‐dependent optical constants and complex conductivity were extracted using Kramers–Kronig transformations (KKT) and validated by TDTS. Despite stronger carrier localization, TTC‐DTO exhibits higher intrinsic conductivity due to increased carrier density, whereas TTC‐PD shows lower conductivity but higher mobility arising from more delocalized transport pathways. This conceptual study demonstrates the potential of FDTS and TDTS as a combined and complementary platform for comprehensive analysis of the ground state charge transport properties of frameworks across the extended THz regime.

## Graphical abstract

This work reports a combined frequency‐domain and time‐domain terahertz (THz) spectroscopic approach to elucidate intrinsic carrier properties and transport mechanisms in framework materials over the extended 0.5–20 THz region. Subtle structural and chemical variations are shown to strongly influence THz charge‐transport behavior across the far–infrared and THz frequency regimes.

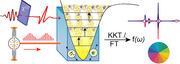

## 1 Introduction

Reticular materials, such as metal–organic frameworks (MOFs) and covalent organic frameworks (COFs), have emerged as a compelling class of optoelectronic materials [1, 2, 3, 4, 5, 6, 7]. Their modular architectures, synthetic tunability, and extended π‐conjugation facilitate control over optical, electrical, and magnetic properties [8, 9, 10, 11, 12, 13, 14]. While traditional contact‐based electrical measurements can provide valuable insights into carrier dynamics and charge transport behavior, they predominantly probe inter‐crystal transport over relatively long length scales under direct‐current conditions [15, 16, 17]. In contrast, THz spectroscopy has evolved into a powerful, non‐invasive approach for interrogating high‐frequency optical conductivity, enabling direct access to short‐range, intra‐crystal charge transport and carrier properties [18, 19, 20, 21, 22, 23, 24].

Beyond conductivity, THz spectroscopy also provides access to the complex refractive index and complex dielectric function, parameters that are essential for advancing fundamental understanding and for evaluating performance in semiconductor technologies, photonic devices, and sensing platforms [25, 26, 27, 28, 29, 30]. In 2018, Dong et al. first reported the use of time‐resolved THz spectroscopy (TRTS) to determine the intrinsic photoconductivity of a two‐dimensional (2D) conjugated MOF, revealing band‐like transport [31]. Subsequently, Yang et al. employed optical pump‐probe THz spectroscopy to investigate the semiconducting behavior of a layered semiconducting MOF magnet [32]. Building on these advances, Fu et al. and others applied TRTS to elucidate excited‐state dynamics and charge transport mechanisms in COFs and MOFs [33, 34, 35]. Recently, Puthukkudi et al. employed TDTS to investigate the ground‐state optical conductivity and carrier properties of two imine‐linked three‐dimensional (3D) COF membranes (COFMs), namely TAM‐DTP and TAM‐DBD [36]. Although TDTS enables direct measurement of intrinsic conductivity and evaluation of charge transport parameters within classical transport models, it is limited by the low acquisition rate of THz transients, which results in reduced spectral resolution. In addition, bandwidth constraints imposed by THz generation and detection mechanisms restrict access to higher‐frequency spectral features, such as excitonic resonances and high‐frequency optical phonon modes. To overcome these challenges, in this work, as proof of concept, we used FTIR‐based FDTS to investigate the steady‐state optical and carrier properties in reticular frameworks (both COF and porous organic polymer (POP)). In FDTS, a broadband thermal source, such as a Hg‐arc lamp, is employed as the excitation source. An interferogram is generated using a Michelson interferometer and subsequently converted into a spectrum via Fourier Transform. This approach enables high spectral resolution over a broad frequency range, extending from the far–infrared to the THz regime (0.5–20 THz), and allows access to low‐energy excitations that may be difficult to resolve with pulsed THz techniques. Since FDTS directly records the frequency‐domain response, the resulting spectra can be readily used to extract optoelectronic parameters [37, 38, 39]. Hence, FDTS offers significant advantages, including ease of operation and the ability to cover a broad spectral region in a single measurement. It is particularly effective for probing low energy vibrational modes, collective lattice dynamics, and intrinsic dielectric responses of materials in the THz frequency range with better frequency resolution compared to TDTS.

For these studies, we selected the imine‐linked COF TTC‐PD as a model crystalline framework synthesized via polycondensation between the tetratopic linker [1,1':4',1''‐terphenyl]‐2',4,4'',5'‐tetracarbaldehyde (TTC) and *p*‐phenylenediamine (PD). The asymmetric geometry of the TTC linker promotes the formation of a closely packed 2D COF backbone with a square–planar grid topology, which is anticipated to facilitate both in‐plane and through‐plane charge delocalization [40]. To systematically examine the influence of linkage chemistry and structural order on charge transport, we synthesized an amorphous counterpart, TTC‐DTO, by condensing TTC with dithoxamide (DTO) to form a thiazolo[5,4‐d]thiazole (TzTz) linkage. The TzTz linkage is known to promote charge stabilization through band dissociation effects [41, 42]. Together, TTC‐PD and TTC‐DTO provide a controlled platform with closely related backbone structures but distinct linkage chemistry and crystallinity, enabling direct comparison of how framework organization and heteroatom incorporation influence low‐frequency carrier dynamics in reticular materials. For comparison, the corresponding molecular analogues Bn‐PD and Bn‐DTO were also prepared and characterized using standard techniques (*vide infra*).

From the FDTS transmittance spectra of TTC‐PD and TTC‐DTO, the absorption coefficient was directly determined. Further on, critical optical parameters, including the extinction coefficient (*k*), refractive index (*η*), real and imaginary parts of the dielectric function (*ε′* and *ε″*), and the real and imaginary parts of the optical conductivity (*σ_r_* and *σ_i_*), were extracted from the absorption data using KKT analysis [38, 43, 44, 45, 46, 47]. To validate the results, the TDTS spectra of TTC‐PD and TTC‐DTO have been acquired using a femtosecond laser‐based spectrometer covering the range of 0.5–2.0 THz. The FFT of the THz transient was in good agreement with the corresponding FDTS spectra. Furthermore, the Drude–Smith (DS) model is applied to disentangle the intrinsic charge transport properties. TTC‐PD and TTC‐DTO, along with their analogues, serve as proof‐of‐concept systems to establish combined and complementary FDTS–TDTS as a general methodology for probing steady‐state carrier dynamics in framework materials, with broad applicability across COFs and related reticular solids. This approach overcomes the spectral limitations of conventional THz techniques and provides a powerful tool for elucidating structure–property relationships in a wide range of advanced materials relevant to next‐generation optoelectronic and electronic devices.

## 2 Results and Discussion

### 2.1 Material Synthesis and Characterization

**FIGURE 1:**
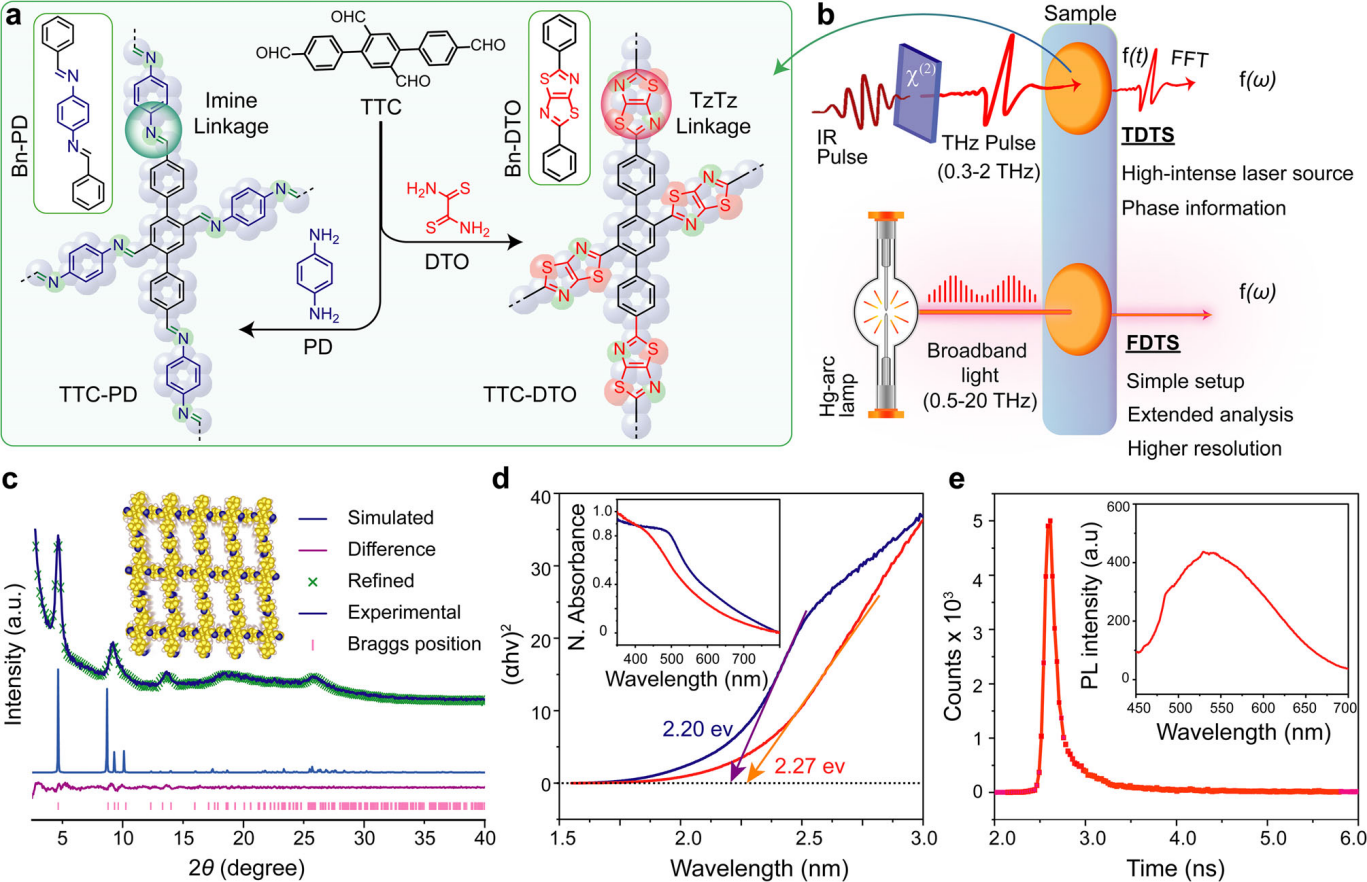
(a) Schematic representation of the synthesis of TTC‐PD and TTC‐DTO, with the chemical structures of their corresponding model molecular analogues Bn‐PD and Bn‐DTO shown as insets; (b) A comparative representation of the combined time‐domain and FTIR‐based frequency‐domain THz spectroscopy approaches, highlighting their features; (c) Powder x‐ray diffraction (PXRD) profile of TTC‐PD with corresponding *Pawley* refinement (*R*_wp_ = 4.83%, *R*_p_ = 3.85%) and the simulated pattern derived from the proposed structural model; (d) Tauc plots of TTC‐PD and TTC‐DTO obtained from their respective UV‐DRS spectra (shown in inset) used to estimate the direct band gaps; 2.20 eV for TTC‐PD (blue) and 2.27 eV for TTC‐DTO (red); (e) Photoluminescence lifetime decay profile of TTC‐DTO (Inset: steady‐state photoluminescence spectrum of TTC‐DTO).

TTC‐PD was synthesized via an acid‐catalyzed Schiff‐base polycondensation between TTC and PD, whereas TTC‐DTO was obtained by condensing TTC and DTO to form a TzTz‐linked framework (Figure 1a, Schemes S1, and S2, ESI). For comparative analysis, two discrete molecular model compounds, Bn‐PD and Bn‐DTO (Figure 1a, Schemes S3, and S4, ESI), corresponding to TTC‐PD and TTC‐DTO, respectively, were also prepared. Detailed synthetic procedures along with nuclear magnetic resonance (NMR) data of the model compounds (Bn‐PD and Bn‐DTO), are provided in Section S2, ESI.

Powder x‐ray diffraction (PXRD) confirmed the crystalline nature of TTC‐PD (Figure 1c, and Figure S5, ESI). The first and most intense diffraction peak, corresponding to the (100) plane, was observed at 2*θ* = 4.7°. Additional reflections appeared at 2*θ* = 9.1°, 13.7°, 18.7°, and 25.8°, which were indexed to the (220), (120), (101), and (001) facets, respectively. Comparison with simulated patterns and Pawley refinement supported an eclipsed (AA) stacking model (*R*_wp_ = 4.83%, *R*_p_ = 3.85%) (Figure S6, ESI). In contrast, TTC‐DTO exhibited no discernible diffraction peaks, indicating its amorphous nature (Figure S7, ESI). Permanent porosity of TTC‐PD and TTC‐DTO was confirmed by N_2_ sorption at 77 K, showing type‐II reversible isotherms with BET surface areas of 336 m^2^ g^−1^ and 224 m^2^ g^−1^ and pore sizes centered at ∼20 Å and ∼19 Å, respectively (Figures S8 and S9, ESI). The chemical compositions of TTC‐PD and TTC‐DTO were confirmed by Fourier transform infrared (FTIR) spectroscopy and ^13^C‐cross polarization magic angle spinning (CP‐MAS) solid‐state NMR spectroscopy (Section S5, ESI), while their thermal stability and morphology were investigated by thermogravimetric analysis (TGA) and electron microscopy (Sections S6 and S7, ESI), respectively.

The electronic band structures of TTC‐PD and TTC‐DTO were investigated using UV–visible diffuse reflectance (UV‐DRS) spectroscopy (Figure 1d). Both TTC‐PD and TTC‐DTO exhibited broad absorption in the range of 400–550 nm, and analysis of the corresponding Tauc plots yields direct optical band gaps of 2.10 eV and 2.27 eV, respectively, confirming their semiconducting nature (Figure 1d). The slightly narrower band gap of TTC‐PD is attributed to extended π‐conjugation through the imine linkage, whereas the TzTz linkage in TTC‐DTO introduces more localized electronic states. Photoluminescence (PL) measurements performed in isopropyl alcohol (IPA) further revealed the contrasting excited‐state behaviors of the two materials. TTC‐PD is essentially nonemissive, consistent with efficient charge separation and dominant nonradiative decay pathways. In contrast, TTC‐DTO showed pronounced fluorescence with an emission peak centered at 529 nm with a photoluminescence lifetime of 0.76 ns, indicative of localized excitonic recombination (Figure 1e). The relatively flexible imine linkage in TTC‐PD likely facilitates intramolecular motions that enable efficient nonradiative relaxation, thereby quenching luminescence. Conversely, the rigid and planar TzTz linkage in TTC‐DTO restricts these intramolecular motions, suppressing nonradiative pathways and favoring radiative recombination.

**FIGURE 2:**
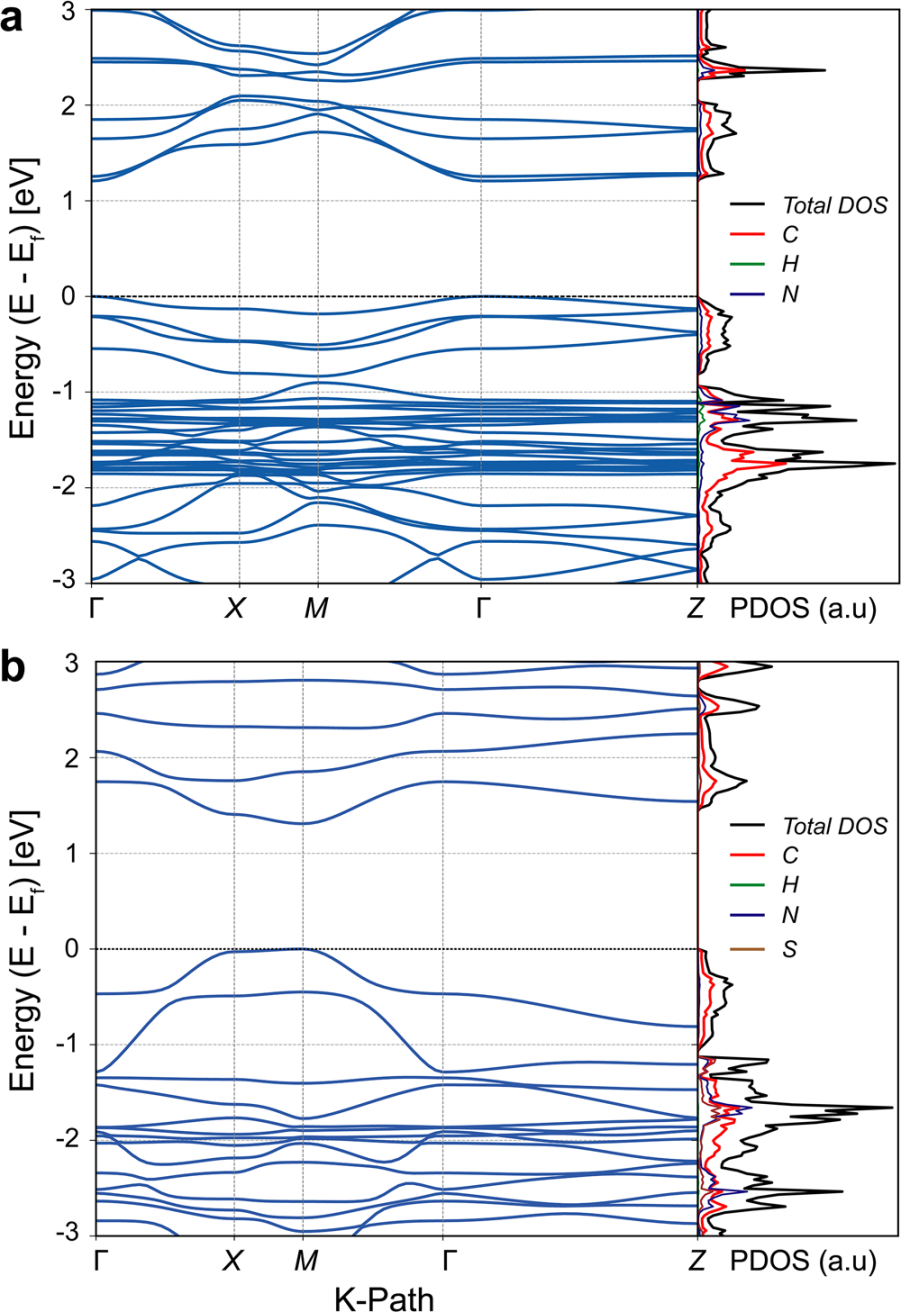
Theoretically calculated electronic band structures of (a) TTC‐PD and (b) TTC‐DTO, obtained from DFT calculations on multilayer idealized crystalline models, together with their corresponding projected densities of states (PDOS). These calculations represent the intrinsic electronic structure and upper limits of charge transport in locally ordered framework domains.

For deeper insight, we further computed the electronic band structures of multilayer idealized models of TTC‐PD and TTC‐DTO employing DFT (Figure 2, Section S8, and Figure S20, ESI). The calculated fundamental band gaps, 1.21 eV for TTC‐PD and 1.31 eV for TTC‐DTO, are in qualitative agreement with the experimentally observed trends (Figure 2). As typically observed in DFT‐based calculations, the absolute values are underestimated owing to the limitations of the generalized gradient approximation (GGA) exchange‐correlation functional used [33, 34]. Further analysis of the band dispersions revealed pronounced anisotropy. TTC‐PD exhibits relatively uniform, light electron masses along Γ→X and Γ→M directions (m^*^_e_ ∼0.385 *m_0_*), indicative of delocalized electronic states. In contrast, TTC‐DTO shows heavier and more direction‐dependent electron effective mass (m^*^_e_ ≈ 1.01–1.16 *m_0_*) and strongly anisotropic hole effective mass (0.60–3.49 m_0_) (Tables S1 and S2, ESI). These features highlight the influence of chemical backbone and linkage chemistry on carrier delocalization, with TTC‐PD favoring more isotropic transport and TTC‐DTO showing pronounced directional confinement.

It is important to note that these band structure calculations are based on idealized crystalline models and therefore represent the intrinsic electronic structure and upper limits of charge transport capabilities. In practice, the amorphous nature of synthesized TTC‐DTO precludes coherent band transport, with charge transport occurring predominantly via hopping mechanisms. Consequently, these calculations serve as a benchmark for the maximum attainable transport performance within locally ordered domains of the semiconducting material.

### 2.2 Optoelectronic Properties and Charge Transport Analysis in the THz Regime

**FIGURE 3:**
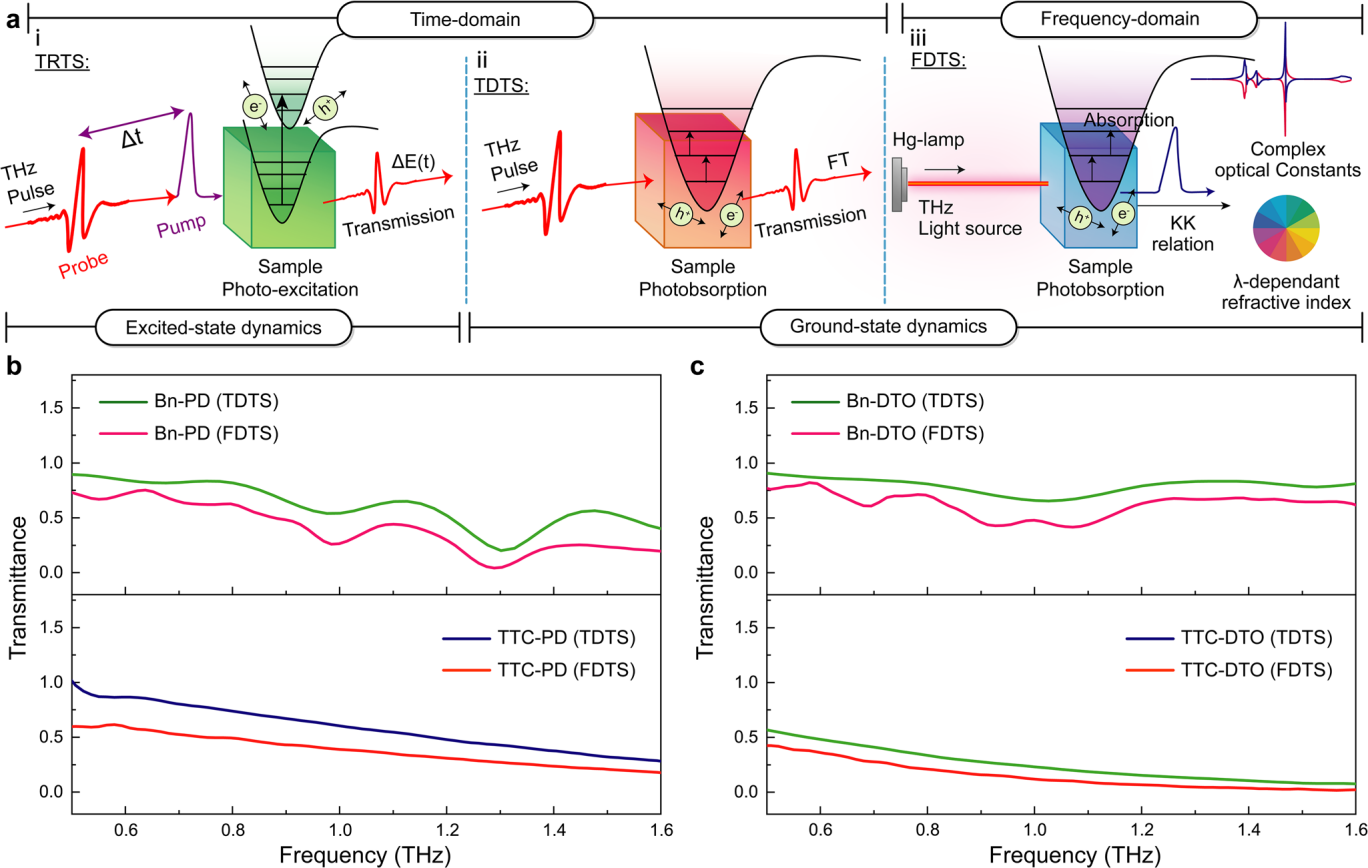
(a) Various modes of THz spectroscopy; i) Time‐resolved THz spectroscopy (TRTS); ii) Time‐dependent THz spectroscopy (TDTS); and iii) FT‐IR based frequency‐dependent THz spectroscopy (FDTS). (FT: Fourier transform; KK: Kramers–Kronig); (b) and (c) Comparison of the THz transmittance spectra of TTC‐PD and TTC‐DTO, along with their molecular analogues Bn‐PD and Bn‐DTO in the range of 0.5–1.6 THz.

To understand variations in optoelectronic properties across the THz spectral range, a combined and complementary approach of TDTS and FDTS is used (Figure 1b). Figure 3a depicts the various modes of THz spectroscopy. FDTS spectra of TTC‐PD and TTC‐DTO were measured in a Bruker Vertex 70 V FTIR spectrometer operated under vacuum. To cover the extended range, the FDTS spectra were acquired over two spectral regions: 33–680 cm^−1^ (corresponding to 1–20 THz) and 16–55 cm^−1^ (∼0.5–1.6 THz). TDTS measurements were performed using the same pellets (Table S3, ESI) in the range of 0.5–2 THz. In TDTS, the reference signal exhibited the highest amplitude across the entire frequency range (Figure S21, ESI). Among the samples, TTC‐PD transmitted a greater fraction of the incident THz radiation than TTC‐DTO, particularly at frequencies above 1 THz. For both samples, the amplitude decreased with increasing frequency, consistent with enhanced absorption and/or scattering at higher energies. The optoelectronic properties were directly extracted by analyzing the phase difference between the sample and the reference.

**FIGURE 4:**
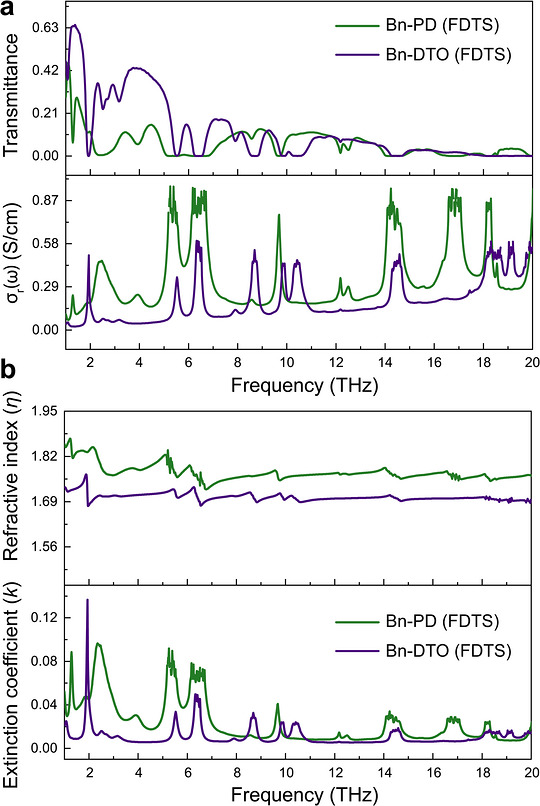
Optoelectronic properties of Bn‐PD and Bn‐DTO in the extended THz region (1–20 THz). (a) Comparison of the transmittance spectra of molecular analogues Bn‐PD and Bn‐DTO along with the real part of their optical conductivity. Both molecular analogues show different vibrations; (b) Comparison of refractive index and extinction coefficient of Bn‐PD and Bn‐DTO. The variation of *η* for Bn‐PD and Bn‐DTO is from 1.86 to 1.80 and 1.76 to 1.69, respectively. The variation of k for both compounds is from 0.018 to 0.096 and 0.005 to 0.133, respectively.

The transmission spectra of TTC‐PD and TTC‐DTO, along with their molecular analogues Bn‐PD and Bn‐DTO, are shown in Figure 3b and c in the range of 0.5–1.6 THz. The molecular analogues exhibit higher overall transparency in the THz region, accompanied by distinct vibrational features, the assignments of which are discussed in detail later in this manuscript. However, transitioning from discrete molecules to extended framework materials, such as TTC‐PD and TTC‐DTO, introduces additional intermolecular interactions and collective vibrational modes, resulting in a continuous decrease in transmittance across this frequency range. Despite this reduction, both framework materials remain highly transparent at frequencies below 1 THz, making them suitable for THz optical components such as lenses and windows [48, 49]. Therefore, TTC‐PD and TTC‐DTO offer different functional advantages compared to discrete molecules, with suitability depending on the targeted THz application and operational frequency window. A similar trend is observed in the extended spectral region from 1 THz to 20 THz. In this range, the transmittance spectra of Bn‐PD and Bn‐DTO display well‐resolved vibrational features (Figure 4a). TTC‐PD and TTC‐DTO exhibit markedly reduced transmittance in the extended region due to the increased optical density. To overcome this issue, we measured the transmittance spectra of both samples using an ATR module, and discussed in Section S9, ESI (Figure S22, ESI).

The real part of the refractive index (*η*) for TTC‐PD and TTC‐DTO decreases modestly across the 0.5–1.6 THz range, varying from 1.45 to 1.36 and 1.60 to 1.50, respectively (Figure S24a,b, ESI). This reveals that the TTC‐DTO has consistently higher *η* than TTC‐PD throughout the spectral range. This trend can be attributed to the higher atomic polarizability of sulfur compared to nitrogen, arising from its larger atomic size, less shielding effect [50, 51]. As the refractive index is fundamentally governed by atomic and molecular polarizability, increased ease of electron‐cloud distortion leads to stronger interaction with the incident THz field and, consequently, a higher *η* [52]. The variation of *η* for Bn‐PD and Bn‐DTO is from 1.80 to 1.70 and 1.71 to 1.69, respectively, in the same range (Figure S24a,b, ESI), suggesting that upon polymerization of the molecular analog to framework materials, the *η* decreases. The variation of *η* for Bn‐PD and Bn‐DTO is from 1.86 to 1.80 and 1.76 to 1.69, respectively in the range 1–20 THz (Figure 4b). This value is higher than in the previous lower frequency range. This is because at higher frequencies, the various polarization mechanisms (the movement of electron clouds and atomic nuclei) can respond more readily and in phase with the rapidly oscillating electric field, leading to a stronger interaction and a higher refractive index.

Beyond transmittance and refractive index, a comprehensive analysis of additional THz optical parameters‐including the real and imaginary parts of the dielectric function (*ε′* and *ε″*), extinction coefficient (*k*), and absorption coefficient (*α*) was carried out for both framework materials and their corresponding molecular analogues (Table S4, ESI). Across the 0.5–1.6 THz region, as well as the extended 1–20 THz window, all these parameters exhibit trends that are fully consistent with the transmittance and refractive index behavior (Figure 4, and Table S4, ESI). In particular, sulfur‐containing TTC‐DTO consistently shows enhanced dielectric response and stronger interaction with the THz field compared to TTC‐PD, reflecting the higher atomic polarizability of sulfur relative to nitrogen. Conversely, reticulation from discrete molecular units to extended covalent frameworks leads to an overall reduction in dielectric constant and absorption, indicative of suppressed localized vibrational contributions in the rigid framework architecture. These observations underscore how linker chemistry and framework formation jointly govern low energy electromagnetic response in reticular materials. A detailed frequency‐dependent discussion of *ε′*, *ε″k*, and *α* together with supporting spectra and quantitative analysis, is provided in the Supporting Information (Figures S23–S26, and Table S4, ESI) [53, 54, 55, 56]. To confirm measurement reproducibility, FDTS spectra for each sample were acquired three times, yielding nearly identical transmittance within the signal‐to‐noise ratio, thereby confirming high measurement reliability (Figure S27, ESI). A quantitative comparison of the FDTS and TDTS transmission spectra shows good agreement (*R* ^2^ ≈ 0.99, RMSE < 1%), while the Bland–Altman plots reveal no frequency‐dependent bias, with the small deviations being predominantly systematic, thereby validating the reliability of the FDTS (Figure S28, ESI).

Furthermore, the THz optical conductivity is a frequency‐dependent complex response to an oscillating electric field, comprising a real (*σ_r_(ω*)) and an imaginary (*σ_i_(ω)*) component. The real part represents dissipative charge transport, while the imaginary part reflects the nondissipative, polarization‐driven response of the material. A negative *σ_i_(ω)* corresponds to a capacitive response associated with localized charges, vibrational modes, and bound electronic states, whereas a positive *σ_i_(ω)* indicates inductive behavior typical of free‐carrier transport in metals [57]. In the 0.5–1.6 THz range, *σ_r_(ω)* for TTC‐PD and TTC‐DTO varies from 0.03–0.10 and 0.04–0.16 S cm^−1^, respectively (ESI), while Bn‐PD and Bn‐DTO show values of 0.03–0.25 and 0.02–0.05 S cm^−1^ (Figure S23e,f, ESI), increasing further in the extended 1–20 THz region (Figure 4a). Notably, Bn‐PD exhibits higher *σ_r_(ω)* than TTC‐PD, whereas Bn‐DTO shows lower *σ_r_(ω)* than TTC‐DTO in the low‐frequency regime. The imaginary component *σ_i_(ω)* is negative for all materials, with TTC‐DTO (−0.44 to −1.10 S cm^−1^) displaying consistently more negative values than TTC‐PD (−0.31 to −0.77 S cm^−1^) over 0.5–1.6 THz (Figure S25c,d), indicative of a stronger capacitive response arising from the higher polarizability of sulfur. Molecular analogues exhibit larger |*σ_i_(ω)*| than the corresponding frameworks, and |σ_i_(ω)| increases further in the 1–20 THz range (Figures S26d, ESI), highlighting the dominance of localized polarization processes in discrete molecules compared to extended frameworks.

To further elucidate charge transport characteristics in TTC‐PD and TTC‐DTO, we analyzed the complex THz conductivity using the Drude–Smith (DS) model (Figure S29, ESI). The DS model allowed the extraction of key electronic parameter *viz*., scattering time (*τ*), plasma frequency (*ω_p_*), carrier density (*n*), backscattering parameters (*c_1_*), and DC mobility (*μ_DC_*) (Table S5, ESI). Here, the DS model is employed as a phenomenological framework to quantify the degree of carrier localization and backscattering rather than as a strict microscopic description of band transport. The parameters obtained from the DS fitting were estimated using the effective mass (m*) obtained from DFT band structure calculations. We note that in real materials the effective mass may vary with factors such as crystallinity, structural disorder, temperature etc. Therefore, the reported carrier concentration values should be considered as approximate estimates; however, reasonable variations in m* would primarily influence the absolute magnitude of n without affecting the comparative trends observed between the different materials. Within this framework, the carrier scattering time of TTC‐PD (0.063 ps) is longer than TTC‐DTO (0.0487 ps), suggesting TTC‐PD has lower energetic barriers and smoother pathways for the carriers. TTC‐DTO (0.214) shows nearly tenfold higher *ω_p_* compared to TTC‐PD (0.123), which shows the same trend as the *c_1_*. The *c_1_* of TTC‐PD is −0.866 and TTC‐DTO is −0.957, inferring that TTC‐PD shows partial localization, while TTC‐DTO shows strong localization, that is, TTC‐DTO has trapped carriers. The extracted scattering time should be interpreted here as an effective momentum relaxation time rather than a microscopic band‐scattering time. The combined effects of carrier masses, scattering time, and localization are reflected in the DC mobilities. TTC‐PD has much higher *μ*_DC_ (38 cm^2^/Vs), nearly three times that of TTC‐DTO (13.8 cm^2^/Vs). Hence, the TTC‐PD supports more efficient long‐range transport carrier, due to combination of lighter mass, longer scattering time, and more delocalization through the imine linkage (Tables S5 and S6, ESI). On the other hand, although TTC‐DTO possesses a higher carrier density, these carriers are confined within a strongly localized environment. TTC‐PD shows lower conductivity but higher mobility, because it supports fewer charge carriers that are more delocalized due to weaker backscattering and longer scattering time. In contrast, TTC‐DTO exhibits a higher real part of the THz conductivity (0.04–0.16 S cm^−1^) compared to TTC‐PD (0.03–0.10 S cm^−1^) in the 0.5–1.6 THz range, despite its lower mobility. This apparent contradiction arises from the significantly higher carrier density in TTC‐DTO, as evidenced by its larger plasma frequency, which enhances *σ_r_(ω)*. Simultaneously, strong carrier localization and pronounced backscattering suppress the net drift velocity and hence the mobility.

Furthermore, to decouple the effects of structural order and linkage chemistry, we investigated a chemically equivalent amorphous analogue of TTC‐PD, denoted as TTC‐PD (amor) (Section S10, ESI). DS analysis of the complex conductivity of TTC‐PD (amor) reveals that the amorphous phase exhibits a shorter carrier scattering time (*τ* ≈ 0.019 ps) and a highly negative backscattering parameter (*c*_1_ ≈ −0.99), indicating pronounced carrier localization and nearly complete backscattering, consistent with a disordered transport regime (Figure S32 and Table S7, ESI). The fitted *c*_1_ value for the crystalline sample deviates from the strongly localized limit, reflecting comparatively weaker backscattering and more delocalized transport pathways within the ordered framework. This is in line with the conductivity trends, where TTC‐PD (amor) exhibits a more negative imaginary conductivity and stronger localization effects compared to TTC‐PD. The comparative DS analysis confirms that amorphization leads to enhanced disorder‐induced carrier localization, whereas the crystalline framework supports comparatively more coherent and mobile charge transport. In comparison, TTC‐DTO shows a similarly localized behavior, indicating that while the TzTz linkage contributes to carrier confinement, the absence of crystallinity is possibly the primary factor contributing to carrier localization in these systems.

To gain further insight into how the charge transport behavior evolves with temperature in these framework materials, we performed temperature‐dependent TDTS measurements (60–120°C) (Section S11, ESI). The DS analysis of the temperature‐dependent complex conductivity data revealed that in both TTC‐PD and TTC‐DTO, the scattering time (*τ*) decreases with increasing temperature, possibly due to enhanced carrier‐phonon interactions, while the plasma frequency (*ω*_p_) increases, indicating thermal activation of charge carriers (Figure S33 and Tables S8–S11, ESI). Notably, TTC‐DTO exhibits a higher rise in *ω*_p_ compared to TTC‐PD, and overall higher carrier density. Despite this, TTC‐PD consistently shows longer scattering time and significantly higher DC mobility, indicating more efficient charge transport in TTC‐PD. The backscattering parameter (*c*_1_) remains strongly negative and relatively insensitive to temperature in both systems, confirming the persistence of carrier localization. However, *c*_1_ is consistently more negative in TTC‐DTO, indicating stronger localization and enhanced backscattering in the amorphous system. These results suggest that while thermal activation increases the number of participating carriers in both systems, the nature of transport remains fundamentally different. Consequently, while both systems follow hopping‐dominated transport, TTC‐DTO is characterized by higher carrier density but more localized carriers, whereas TTC‐PD exhibits comparatively weaker localization and more coherent carrier motion, resulting in higher mobility and more efficient transport pathways. Temperature‐dependent FTIR measurements show no observable changes with increasing temperature, confirming the structural and chemical integrity of the material (Figure S34, ESI). Additionally, temperature‐dependent TDTS measurement of TTC‐PD (amor) shows an overall increase in *σ_r_(ω)* with increasing temperature, indicating thermally activated carrier participation (Figure S35; Tables S12 and S13, ESI). However, *σ_i_(ω)* remains strongly negative with only modest variation, reflecting persistent carrier localization. Within the DS framework, this behavior corresponds to short scattering times and strong backscattering (*c_1_* ∼ −1), consistent with a highly disordered transport regime. Compared to crystalline TTC‐PD, which shows a reduction in the magnitude of *σ_i_(ω)* with temperature, TTC‐PD (amor) exhibits a much weaker response, indicating the dominant role of structural disorder in limiting charge transport. The similarity between TTC‐PD (amor) and TTC‐DTO further shows that strong localization primarily arises from the absence of structural ordering, while linkage chemistry plays a secondary role.

For TTC‐DTO and TTC‐PD (amor), which are amorphous in nature, DFT calculations based on an idealized structural analogue are used solely to provide qualitative insight into local electronic coupling and effective mass trends. Furthermore, the underlying transport mechanism is completely insensitive to the assumed mass. As the DS model parameters (*τ*, *c*_1_, *ω*_p_) are determined directly from the conductivity spectra and do not depend on the assumed effective mass, changing m* only rescales the mobility without altering the fundamental transport behavior (Tables S6, S9, S11, and S13, ESI). Thus, the mechanism remains identical while only the absolute mobility shifts. By successfully broadening the experimental window from the THz to the far–IR region, we have captured a more comprehensive picture of the frequency‐dependent optical behavior of these materials. This broader spectral analysis provides deeper insight into low‐frequency dielectric dynamics and vibrational modes that are otherwise inaccessible within the conventional TDTS limits.

### 2.3 Analysis of Absorption Features in Low THz Frequencies

**FIGURE 5:**
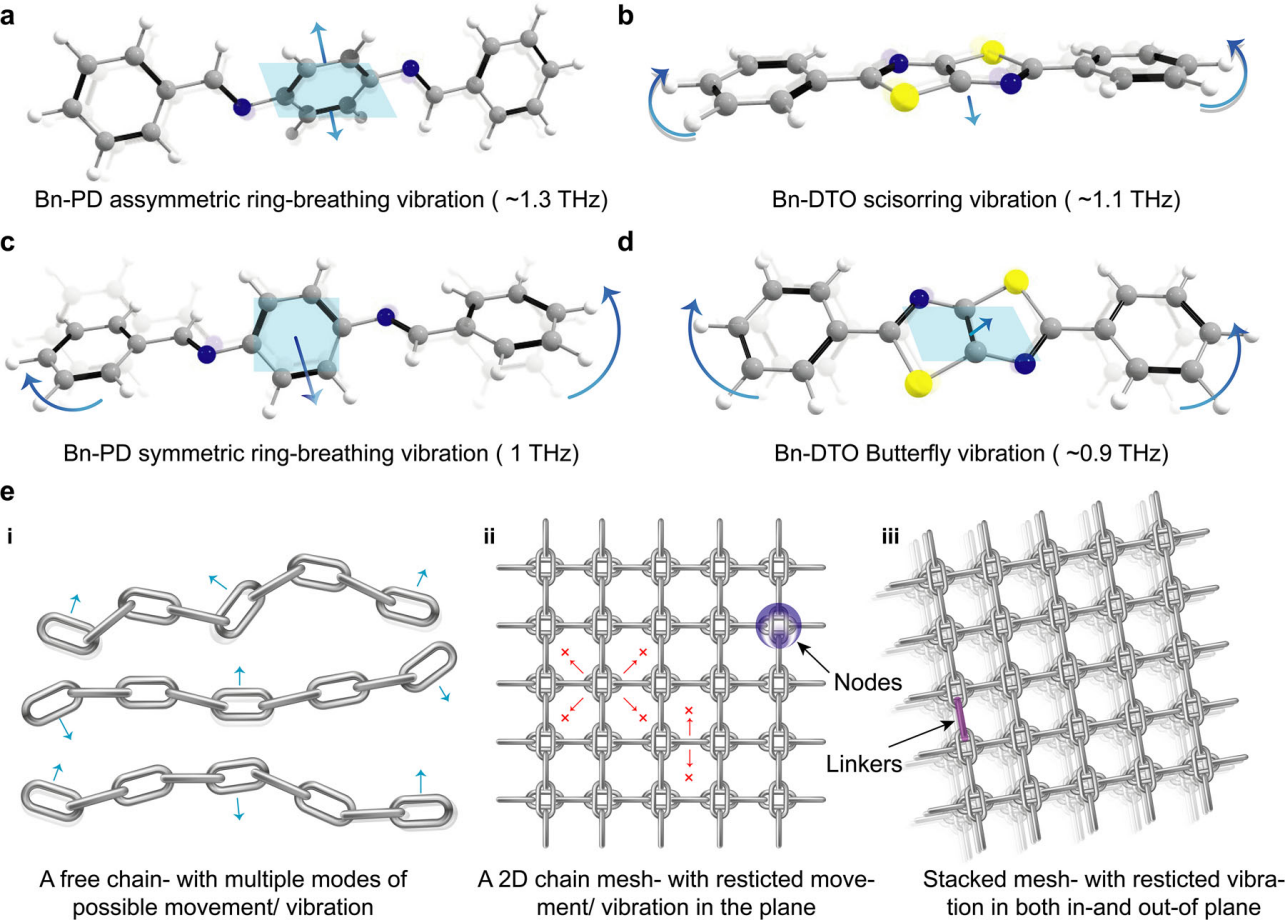
(a), (b) Different vibrational peaks observed for Bn‐PD in the THz region. The two distinct peaks at 0.98 (symmetric ring breathing motion) and 1.28 THz (asymmetric ring breathing motion) of Bn‐PD in the THz region; (c), (d) Three distinct peaks at 0.68 (ring breathing motion), 0.98 (butterfly motion), and 1.07 THz (scissoring motion) of Bn‐DTO in the THz region; (e) A chain‐and‐mesh analogy to describe the arrangements of framework material. Here, a free chain (i), a 2D mesh (ii), and a stacked 2D mesh (iii) respectively represent the molecular analogues, 2D layers of framework materials, and their successive stacking to constitute the framework material.

The molecular analogues Bn‐PD and Bn‐DTO display prominent low‐energy vibrational modes in the THz region, which are conspicuously absent in the framework counterparts. To understand this suppression of THz‐active modes upon reticulation, we set out to first assign the molecular vibrational modes of Bn‐PD and Bn‐DTO, responsible for the observed absorption features in the THz region, by performing DFT calculations (Section S12, ESI) [58, 59]. All geometry optimizations and frequency calculations were carried out using the B3LYP hybrid functional in combination with the 6–311+G(d,p) basis set. The experimental THz transmittance spectra of Bn‐PD and Bn‐DTO revealed distinct absorption peaks, indicative of low‐frequency vibrational modes. In Bn‐PD, two clear absorption peaks were experimentally observed at 0.98 THz and 1.28 THz. Theoretical simulation yielded corresponding vibrational modes at 0.82 THz and 1.00 THz, respectively. The peak at 0.98 THz has been assigned as symmetric ring breathing motion, and the peak at 1.28 THz has been assigned as asymmetric ring breathing motion (Figure 5a,b). Bn‐DTO has three distinct peaks at 0.68, 0.98, and 1.07 THz, with simulated peaks appearing at 0.48, 0.91, and 1.96 THz respectively (Figure 5c,d). The peak at 0.68 THz assigned as the ring breathing motion whereas 0.98 THz is assigned to the butterfly motion. The peak at 1.07 THz has been assigned as the scissoring motion. These are the different vibration modes that contribute to the absorption in the THz region that are observed in the transmittance spectra of Bn‐PD and Bn‐DTO. But for TTC‐PD and TTC‐DTO, there are no observable absorption features in the corresponding region. The possible reason is that upon polymerization to rigid framework structure (TTC‐PD and TTC‐DTO), the monomer flexibility is lost, and local vibration modes (torsions, librations) are restricted due to extended conjugation and network rigidity. Due to its periodic, symmetric lattice structure, vibrational modes may cancel out, leading to a weak or zero net dipole change and hence no distinct THz absorption.

So, the absence of THz absorption peaks in TTC‐PD and TTC‐DTO is due to the loss of local vibrational freedom and the increased lattice symmetry, which suppress dipole‐active transitions. To rationalize this behavior observed in framework materials, a plausible chain‐and‐mesh analogy is proposed (Figure 5e). The molecular analogues, such as Bn‐PD and Bn‐DTO, can be likened to short‐chain segments that retain a high degree of conformational flexibility, allowing low‐frequency vibrational modes (i.e., bending or twisting) along multiple directions. However, when these molecular units are covalently reticulated into an extended 2D network‐akin to interlinking chains into a mesh‐their in‐plane vibrational freedom becomes significantly restricted. Upon further stacking of these 2D sheets into a layered structure, the out‐of‐plane motions are also constrained. This progressive reduction in molecular mobility suppresses low‐energy vibrational modes that would otherwise absorb THz radiation. As a result, the absence of accessible vibrational transitions in the THz frequency range allows THz waves to pass through these materials with minimal attenuation. While the “chain‐and‐mesh” analogy captures the role of rigidification in suppressing localized vibrational modes upon reticulation, additional factors such as symmetry‐induced dipole cancellation and vibrational broadening in the extended framework may also contribute. A quantitative separation of these effects is not accessible from THz spectroscopy alone and lies beyond the scope of the present work.

## 3 Conclusion

In summary, in our maiden attempt, we demonstrate FDTS as an effective and high‐resolution platform for probing steady‐state carrier and vibrational properties, and charge transport in COFs over an extended THz frequency range of 0.5–20 THz. FDTS and TDTS forms a complementary platform for studying ground‐state charge transport in framework materials over an extended THz spectral range. Using a simple new imine‐linked crystalline system, TTC‐PD and TzTz‐linked amorphous counterpart, TTC‐DTO, and molecular analogues (Bn‐PD and Bn‐DTO), we tried to study structure‐property correlations governing carrier density, mobility, and localization in framework versus molecular systems. Frequency‐dependent THz conductivity analysis, interpreted using the DS model, reveals a strong dependence of carrier transport on structural disorder and linkage chemistry. Imine linkages promote greater carrier delocalization and higher mobility, whereas TzTz linkages introduce enhanced carrier trapping despite higher carrier density, leading to distinct ground‐state transport regimes. Temperature‐dependent TDTS reveal that crystalline TTC‐PD exhibits more efficient charge transport than amorphous TTC‐DTO and TTC‐PD (amor), highlighting that structural disorder is the primary factor governing carrier localization, while linkage chemistry plays a secondary role. These findings highlight how subtle chemical connectivity and structural order dictate low‐energy charge transport in COFs. Beyond charge transport, FDTS enables direct access to low‐energy vibrational modes across the far‐infrared and THz regimes. The molecular analogues exhibit characteristic intramolecular vibrations, such as ring‐breathing and symmetric butterfly‐like modes, which are suppressed in the rigid, interconnected framework architectures. This contrast underscores the sensitivity of THz spectroscopy to framework rigidity and collective lattice constraints. By combining a relatively simple experimental setup with high spectral resolution and an expanded analytical window, FDTS may provide a powerful and broadly applicable approach for investigating steady‐state carrier dynamics and vibrational responses in a broad range of molecules and materials.

## Author Contributions

**Satyapriya Nath**: investigation, writing – original draft, data curation, formal analysis, methodology. **Saiprakash Rout**: investigation, writing – original draft, data curation, formal analysis, methodology. **Mahalaxmi Samal**: data curation, methodology, investigation, writing – review & editing, formal analysis, conceptualization. **Snehal Haldankar**: data curation, formal analysis, writing – review & editing. **Md Habib Ahsan**: data curation, formal analysis, writing – review & editing. **Anol Mondal**: data curation, formal analysis, writing – review & editing. **Sudeep Tiwari**: data curation, formal analysis, writing – review & editing. **Adithyan Puthukkudi**: data curation, formal analysis, writing – review & editing. **Avani K V**: data curation, formal analysis, writing – review & editing. **Ashis K. Nandy**: formal analysis, software, writing – review & editing. **Shriganesh S. Prabhu**: supervision, formal analysis, writing – review & editing, methodology, funding acquisition, project administration. **Himansu S. Biswal**: supervision, formal analysis, writing – review & editing, methodology, funding acquisition, project administration. **Xinliang Feng**: supervision, project administration, writing – review & editing, conceptualization, resources, funding acquisition. **Bishnu P. Biswal**: conceptualization, methodology, formal analysis, project administration, supervision, resources, writing – original draft, funding acquisition.

## Conflicts of Interest

The authors declare no conflicts of interest.

## Supporting information




**Supporting File**: The authors have cited additional references within the Supporting Information [60, 61, 62, 63, 64, 65, 66].

## Data Availability

The data that support the findings of this study are available from the corresponding author upon reasonable request.
