# Supplementary material for: Probing Steady‐State Carrier Properties and Charge Transport in Covalent Organic Framework by Frequency‐Domain Terahertz Spectroscopy
Source: Angew Chem Int Ed Engl. 2026 May 1;65(25):e3669544. doi: 10.1002/anie.3669544 (PMC13266929; doi:10.1002/anie.3669544)
Supplement: Supplementary file 1 — Supporting File: The authors have cited additional references within the Supporting Information [60, 61, 62, 63, 64, 65, 66]. [file ANIE-65-e3669544-s001.pdf]

Supporting Information
©Wiley-VCH 2021
69451 Weinheim, Germany

Probing Steady-State Carrier Properties and Charge Transport in Covalent Organic Framework by Frequency-Domain Terahertz Spectroscopy

Satyapriya Nath,[a,b,†] Saiprakash Rout,[a,b,†] Mahalaxmi Samal,[a,†] Snehal Haldankar,[d] Md Habib Ahsan,[b,e] Anol Mondal,[d] Sudeep Tiwari,[d] Adithyan Puthukkudi,[a,b] Avani K V,[a,b] Ashis K Nandy,[b,e] Shriganesh S. Prabhu,[d] Himansu S. Biswal,*[a,b,c] Xinliang Feng,*[f,g] and Bishnu P. Biswal*[a,b,c]

[a]School of Chemical Sciences, National Institute of Science Education and Research (NISER) Bhubaneswar, Odisha 752050, India

[b]Homi Bhabha National Institute, Training School Complex, Anushakti Nagar, Mumbai 400094, India

[c]Center for Interdisciplinary Sciences, National Institute of Science Education and Research (NISER) Bhubaneswar, Odisha 752050, India

[d]Department of Condensed Matter Physics and Materials Science, Tata Institute of Fundamental Research, Mumbai, Maharashtra 400005, India

[e]School of Physical Sciences, National Institute of Science Education and Research (NISER) Bhubaneswar, Odisha 752050, India

[f]Center for Advancing Electronics Dresden (cfaed) & Faculty of Chemistry and Food Chemistry, Technische Universität Dresden, 01062 Dresden, Germany

[g]Max Planck Institute of Microstructure Physics, Halle (Saale) D-06120, Germany

†*These authors contributed equally*

*Email: bp.biswal@niser.ac.in, xinliang.feng@mpi-halle.mpg.de, himansu@niser.ac.in

DOI: 10.1002/anie.2021XXXXX

Table of Contents

Sr. No.	Sections
**S-1**	Experimental Procedures
**S-2**	Synthesis procedures
**S-3**	PXRD, structural modelling and analysis
**S-4**	N_2 sorption studies at 77 K
**S-5**	FT-IR, and ^{13}C CP-MAS solid-state NMR spectra analysis
**S-6**	Thermogravimetric analysis (TGA)
**S-7**	Electron microscopy imaging
**S-8**	Theoretical band-structure Analysis
**S-9**	THz spectroscopy and optical property analysis
**S-10**	Comparison of charge transport properties TTC-PD and TTC-PD (amor)
**S-11**	Temparature-dependant time-domain THz spectroscopy
**S-12**	Theoretical studies
**S-13**	References

Section S-1: Experimental Procedures

Materials:

All commercially available reagents and solvents were used without further purification. Commercially available reagents and starting materials were bought from Sigma-Aldrich, TCI chemicals, and BLD Pharmatech, depending upon their availability.

General instrumentation and methods:

Powder X-ray diffraction (PXRD) patterns were collected at room temperature on a Bruker D8 Advance X-ray powder diffractometer with Cu K_{α} radiation (λ = 1.5418 Å) as the X-ray source. Molecular modelling of the covalent organic frameworks (COFs) was carried out using of BIOVIA Materials Studio, and the structure and unit cell parameters were relaxed using force fields (Forcite, universal force fields with Ewald electrostatic and van der Waals summations method). The unit cells of the model were then refined in the 2*θ* range 2-40° with the experimentally obtained PXRD pattern of COFs in the Reflex module of the BIOVIA Materials Studio, with fixed atom coordinates. The obtained structural models were checked for bond length and bond angle consistency in the structure.

N_2 sorption analyses were performed at 77 K on a Quantachrome Instruments Autosorb iQ MP automatic volumetric instrument. All the samples were outgassed for 12 h at 120 °C under a vacuum prior to the gas adsorption studies. The surface areas were evaluated using the Brunauer-Emmett-Teller (BET) model applied between P/P_0 values of 0.05 and 0.3.

Fourier transform infrared (FT-IR) spectra were recorded using a PerkinElmer FT-IR spectrometer equipped with an attenuated total reflectance (ATR) accessory. The spectra were background corrected and reported with a wave number (cm^{-1}) scale.

Solution NMR (^{1}H-NMR and ^{13}C-NMR) spectra have been recorded in a Bruker advance III 400 MHz Spectrometer.

Solid-state NMR spectra (ssNMR) were recorded on a Bruker Advance III 400 MHz spectrometer (magnetic field 9.4 T). The samples were packed in 4 mm ZrO_2 rotors, which were spun in a Bruker WVT BL4 double resonance MAS probe. The chemical shift was referenced relative to tetramethylsilane (^{13}C) as an external standard. The spinning rate was 12.5 kHz. A standard cross-polarization sequence with a 2-ms ramped contact pulse was used for ^{13}C, and a total of 2K scans were routinely accumulated. Carbon chemical shifts are expressed in parts per million (*δ* scale).

Thermogravimetric analyses (TGA) were carried out on a TG50 analyzer (Mettler-Toledo) and a SDT Q600 TG-DTA analyzer in air at a heating rate of 5 ºC min^{-1} within a temperature range of 30-700 °C.

Elemental analyses were performed in Euro Vector EA 3000 CHNS analyzer.

Scanning electron microscopy (SEM) measurements were executed with a Merlin Compact field effect SEM (FESEM) with a GEMINI-I electron column, Zeiss Pvt. Ltd., Germany. The samples were prepared by sonicating in isopropyl alcohol (IPA) and drop-casting on silicon wafers.

High-resolution transmission electron microscopy (HRTEM) was performed with a JEOL 2100F HRTEM, and FEI TECNAI G2 F20-ST, using an accelerating voltage of 200 kV.

UV-Visible (UV-Vis) spectroscopy was recorded with an Agilent Cary spectrophotometer. The powder samples were dispersed in spectroscopy grade IPA and sonicated prior to the measurement. All measurements were carried out in a quartz cuvette with an optical path length of 1 cm.

Photoluminescence (PL) measurements were carried out on a Cary Eclipse fluorescence spectrophotometer (Agilent Technologies), and the average decay time of the fluorescence species was measured using a time-correlated single photon counting (TCSPC) spectrometer (Edinburgh, model OB920).

Results and Discussion

Section S-2: Synthesis procedures

Synthesis of TTC-PD:

TTC-PD has been synthesized via Schiff base polycondensation reaction between [1,1':4',1''-Terphenyl]-2',4,4'', 5'-tetracarbaldehyde (TTC) and *p*-phenylenediamine (PD). A 10 mL Schlenk tube was charged with TTC (29.21 μmol, 10 mg) and PD (58.42 μmol, 6.32 mg) with a 1 mL solvent mixture of mesitylene: dioxane (1: 1) with 6 M acetic acid (0.1 mL) as the catalyst (Scheme S1). The tube was sealed, sonicated for 15 min, and then degassed by three freeze-pump-thaw cycles. The reaction mixture was heated at 120 ˚C for 3 days. The precipitate formed was collected by filtration and washed with IPA, dimethylacetamide (DMAc), water, and acetone. It was further washed in a Soxhlet apparatus with tetrahydrofuran (THF) for 24 h and then dried under vacuum at 100˚C to obtain TTC-PD as a brown powder in ca. 76 % yield. Anal. Calcd (%) for $C_{34}H_{22}N_4$; C, 83.94; H, 4.56; N, 11.52. Found (%): C, 68.79; H, 4.55; N, 8.51.

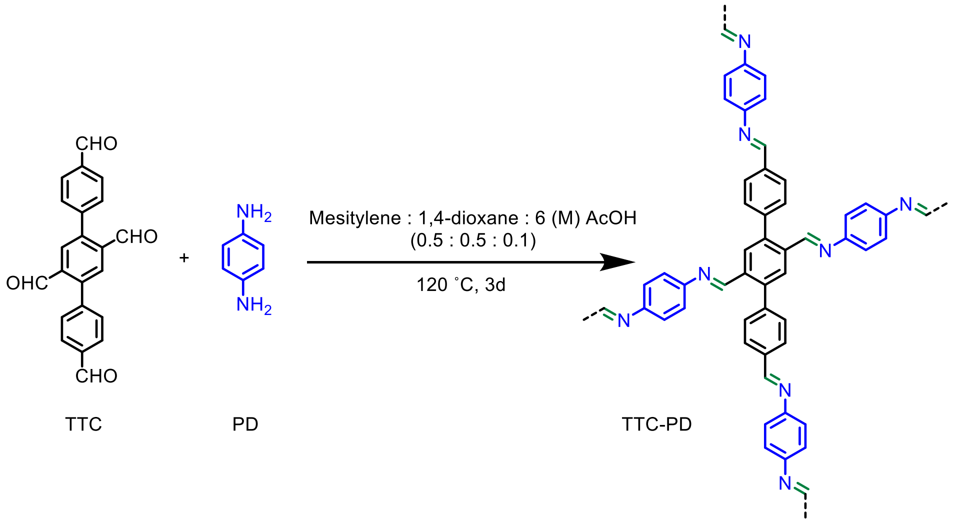

Scheme S1: Schematic representation of the procedure for the synthesis of TTC-PD.

Synthesis of TTC-DTO:

TTC-DTO has been synthesized via a polycondensation reaction between TTC and dithiooxamide (DTO). A 10 mL Schlenk tube was charged with TTC (29.21 μmol, 10 mg) and DTO (6.6 mg, 58.5 μmol) in 1 mL dimethylformamide (DMF). The tube was sealed, sonicated for 15 min, and degassed by three freeze-pump-thaw cycles. The reaction mixture was heated at 150 °C for 3 days. The precipitate formed was collected by filtration and washed thoroughly with DMF, acetone, and THF. It was further washed in a Soxhlet apparatus with THF for 24 h and then dried under vacuum at 100 ˚C to obtain TTC-DTO as a yellow powder in ca. 64 % yield. Anal. Calcd (%) for $C_{26}H_{10}N_4S_4$; C, 61.64; H, 1.99; N, 11.06; S, 25.31. Found (%): C, 61.15; H, 3.93; N, 10.28; S, 5.07.

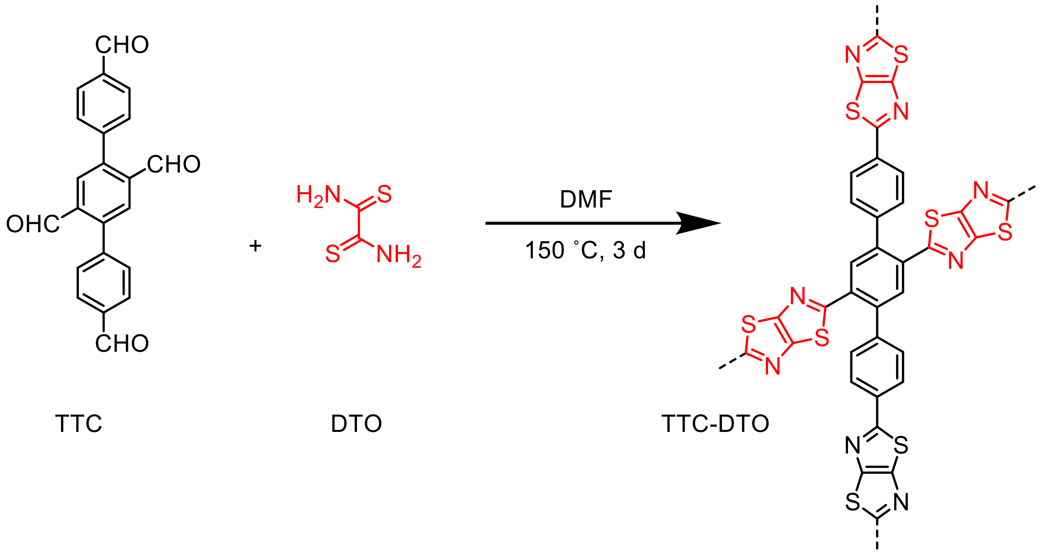

Scheme S2: Schematic representation of the procedure for the synthesis of TTC-DTO.

Synthesis of Bn-PD:

Bn-PD is synthesized following a reported procedure.[60] In a 100 ml round-bottom flask PD (124.7 mg, 2.24 mmol) was dissolved in 20 mL of methanol. To the mixture, benzaldehyde (Bn) (242.23 mg, 4.70 mmol) was added, with immediate formation of precipitate. The reaction mixture was stirred at room temperature for 2h, and the precipitate was filtered out and washed with cold methanol to obtain the product in ca. 90% yield as a yellowish powder. ^{1}H NMR ($CDCl_3$, 400 MHz) δ (ppm) = 8.52 (s, 4H), 7.50-7.48 (m, 6H), 7.30 (s, 4H). ^{13}C {H} ($CDCl_3$, 400 MHz) δ (ppm) = 159.93, 150.14, 136.42, 131.51, 128.95, 121.98.

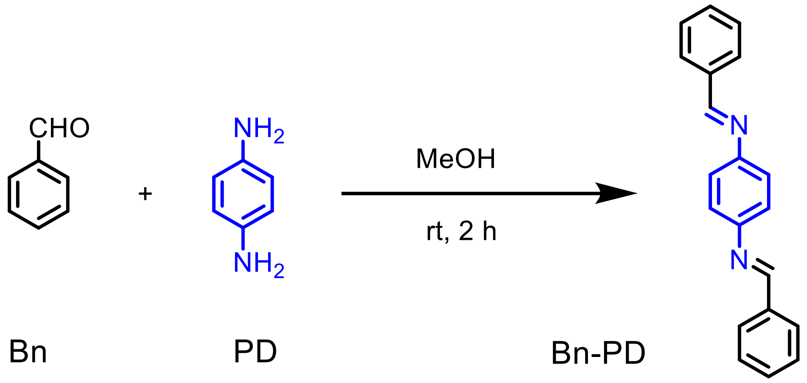

Scheme S3: Schematic representation of the procedure for the synthesis of Bn-PD.

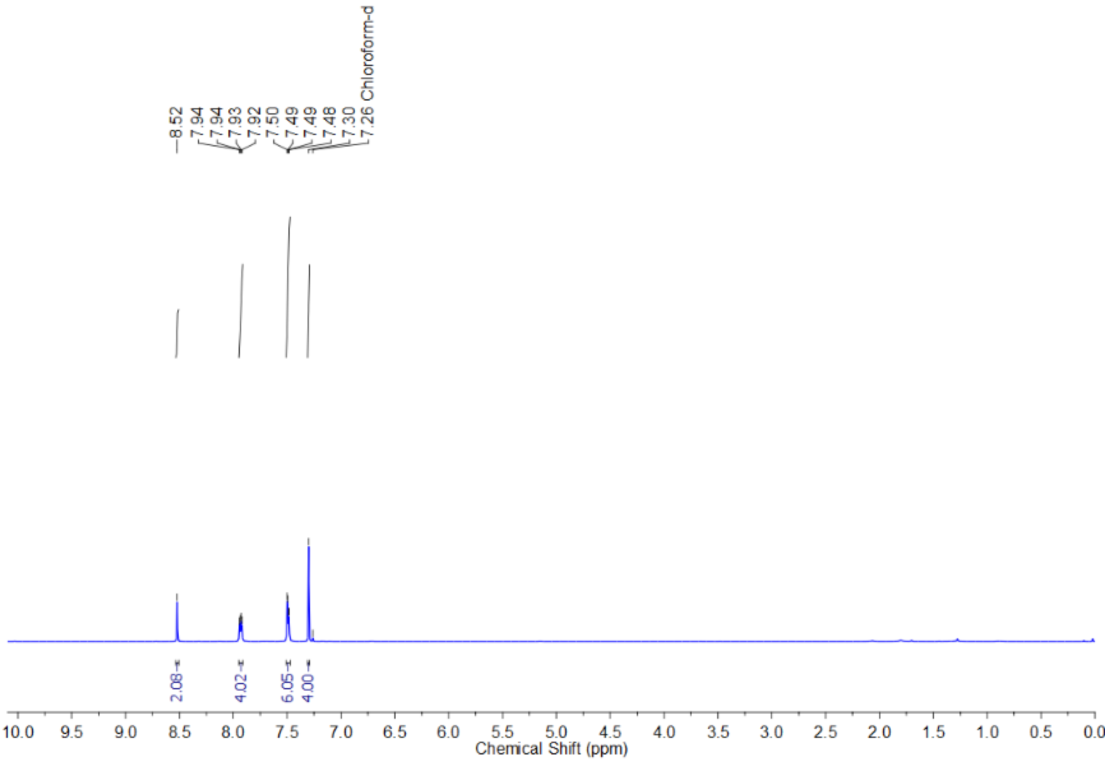

Figure S1: ^{1}H NMR spectra of Bn-PD in chloroform-d (400 MHz).

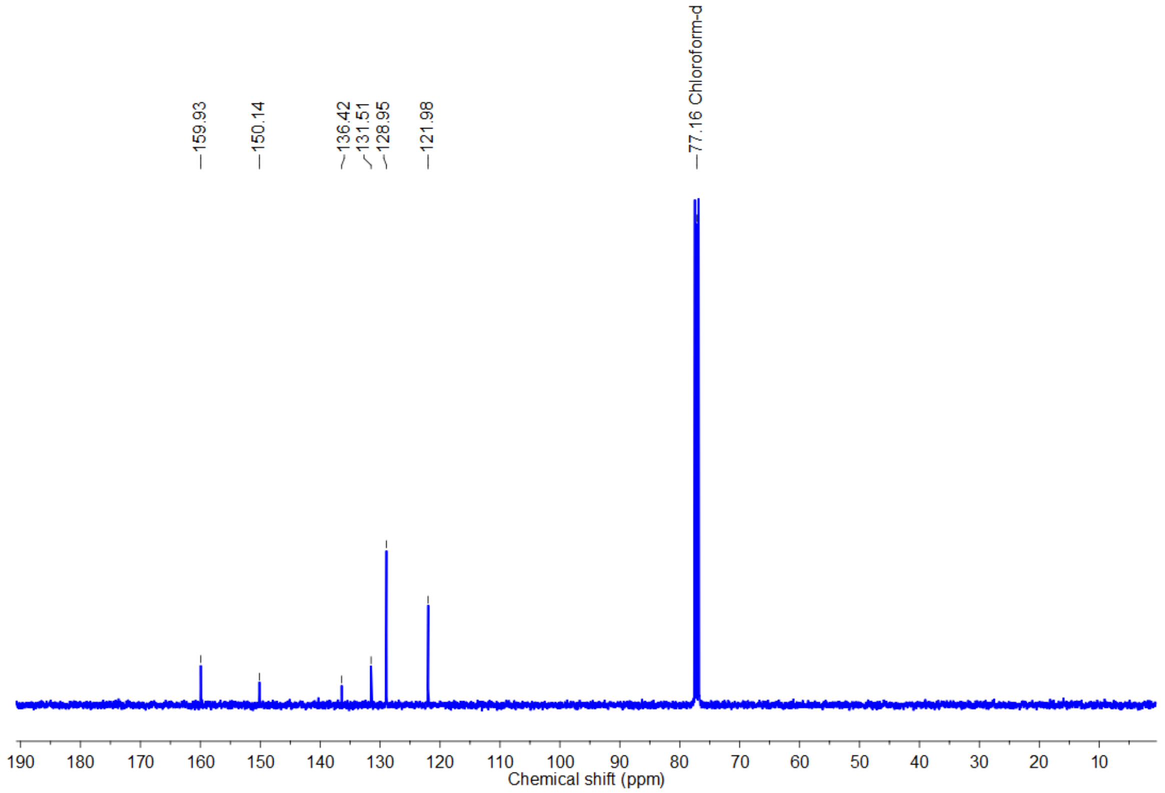

Figure S2: ^{13}C NMR spectra of Bn-PD in chloroform-d (400 MHz).

Synthesis of Bn-DTO:

Bn-DTO is synthesized following a reported procedure.[61] In a 10 mL Schlenk tube, benzaldehyde (Bn) (0.608 mL, 6.0 mmol) and dithiooxamide (DTO) (361 mg, 3.0 mmol) were taken with 10 mL DMF under N_2 atmosphere. The tube was sealed and heated at 150 °C for 24 h. The reaction mixture was cooled down, and the obtained precipitate was collected by filtration, washed with diethyl ether, and recrystallized from dichloromethane to obtain Bn-DTO as a pale-yellow powder in ca. 60% yield. ^{1}H NMR ($CDCl_3$, 400 MHz) δ (ppm) = 8.02–8.00 (m, 4H), 7.49-7.47 (m, 6H). ^{13}C {H} ($CDCl_3$, 400 MHz) δ (ppm) = 169.35, 134.14, 130.84, 129.30, 126.59.

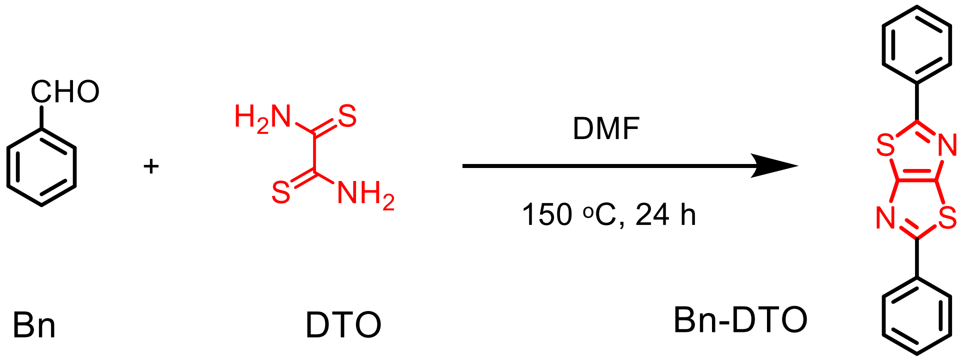

Scheme S4: Schematic representation of the procedure for the synthesis of Bn-DTO.

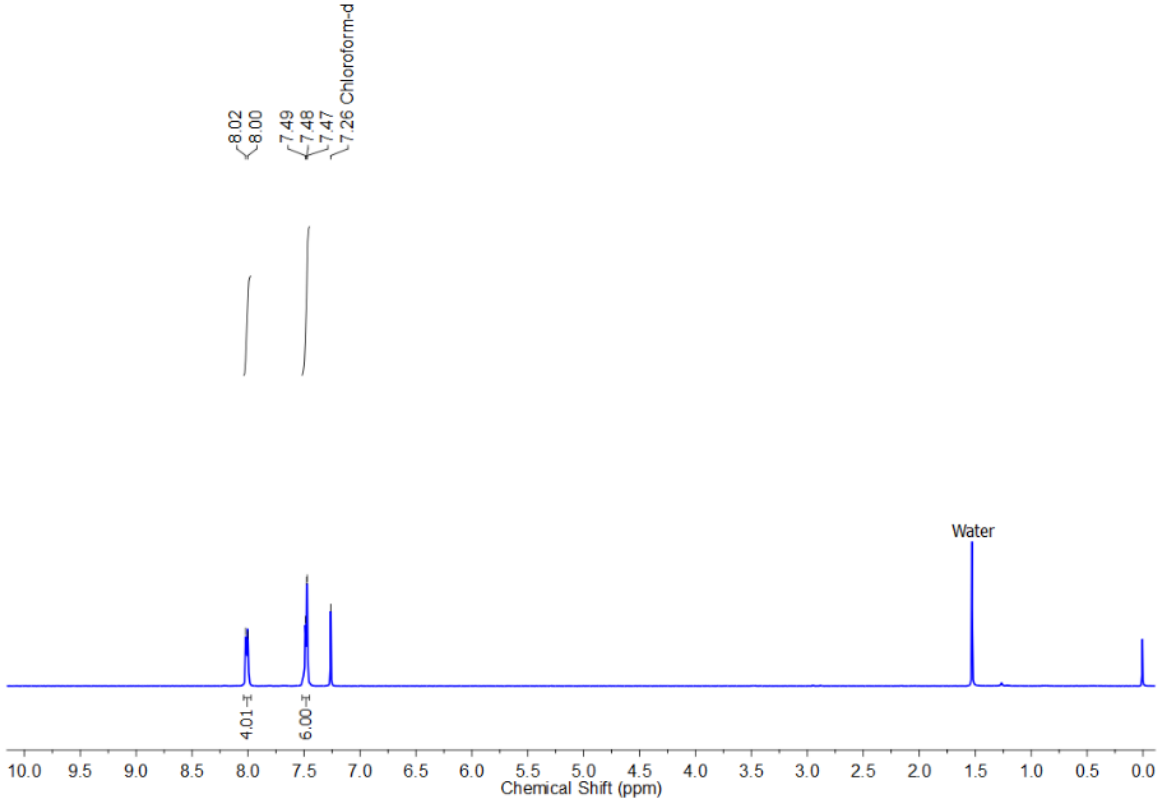

Figure S3: ^{1}H NMR spectra of Bn-DTO in chloroform-d (400 MHz).

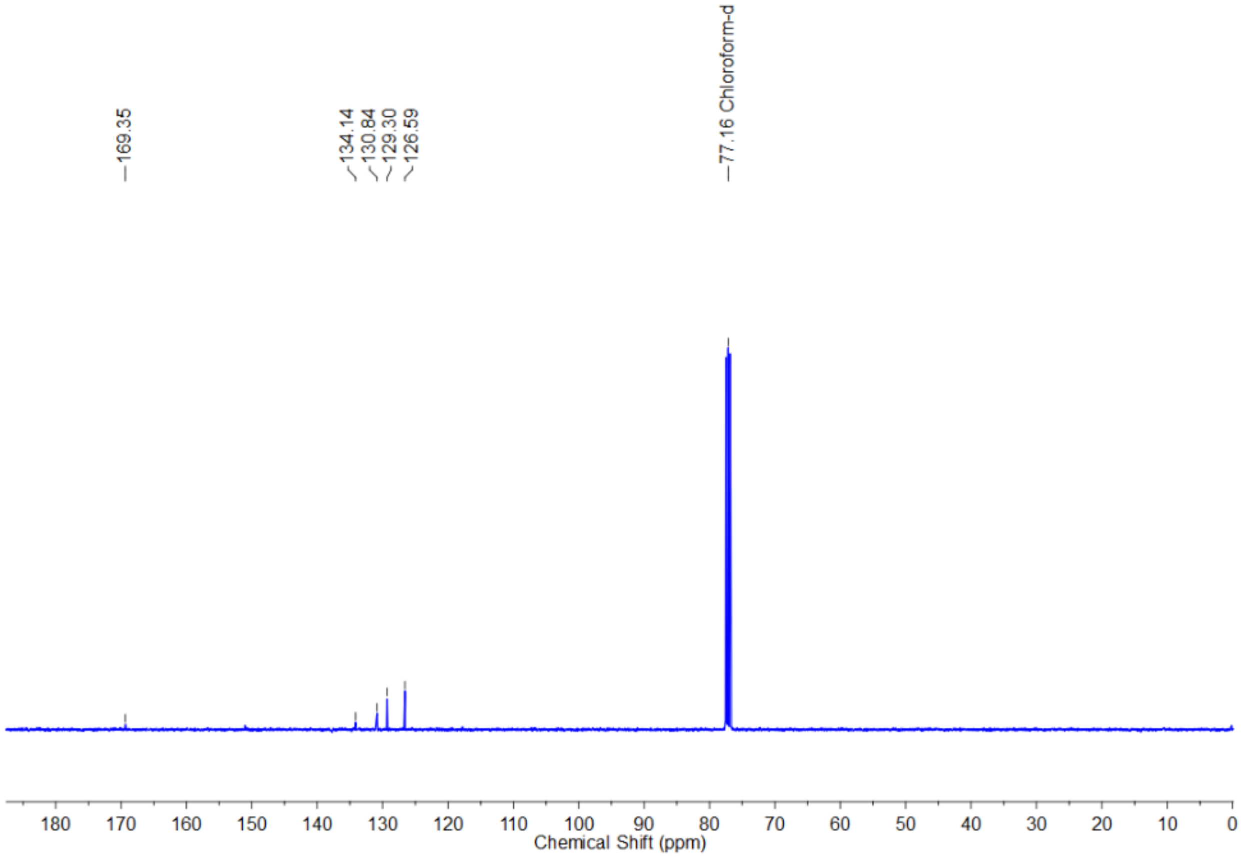

Figure S4: ^{13}C NMR spectra of Bn-DTO in chloroform-d (400 MHz).

Section S-3: PXRD, structural modelling and analysis

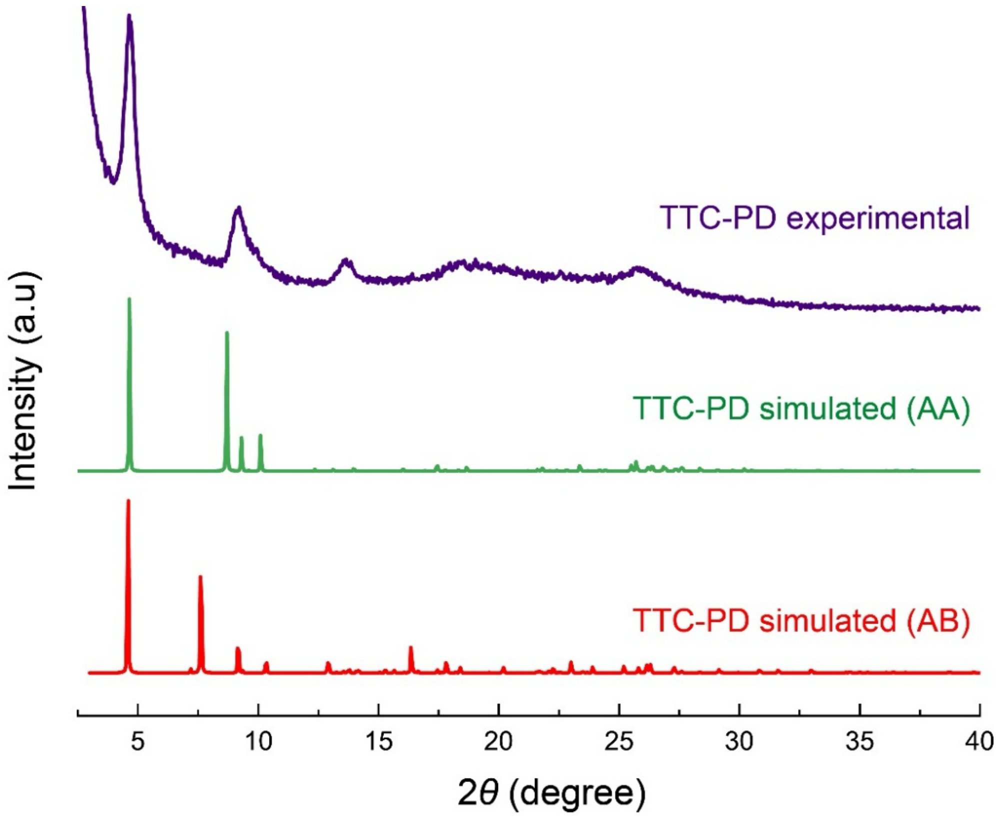

Figure S5: Simulated (AA and AB stacking) and experimental PXRD pattern of TTC-PD COF.

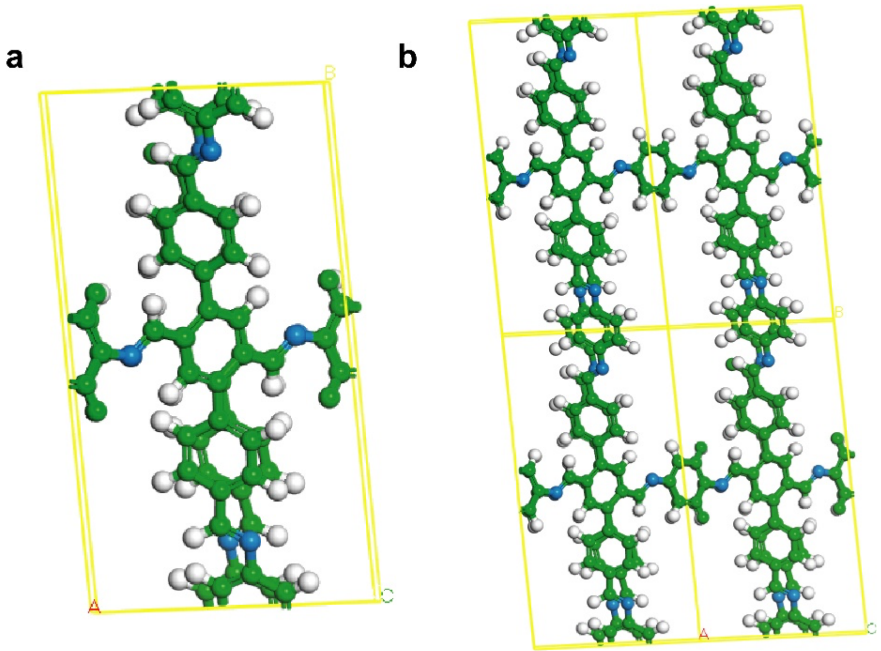

Figure S6: Structural modelling of TTC-PD. a) The optimized unit cell and b) a 2×2 lattice unit of TTC-PD. Atom representation: green: C; blue: N; white: H.

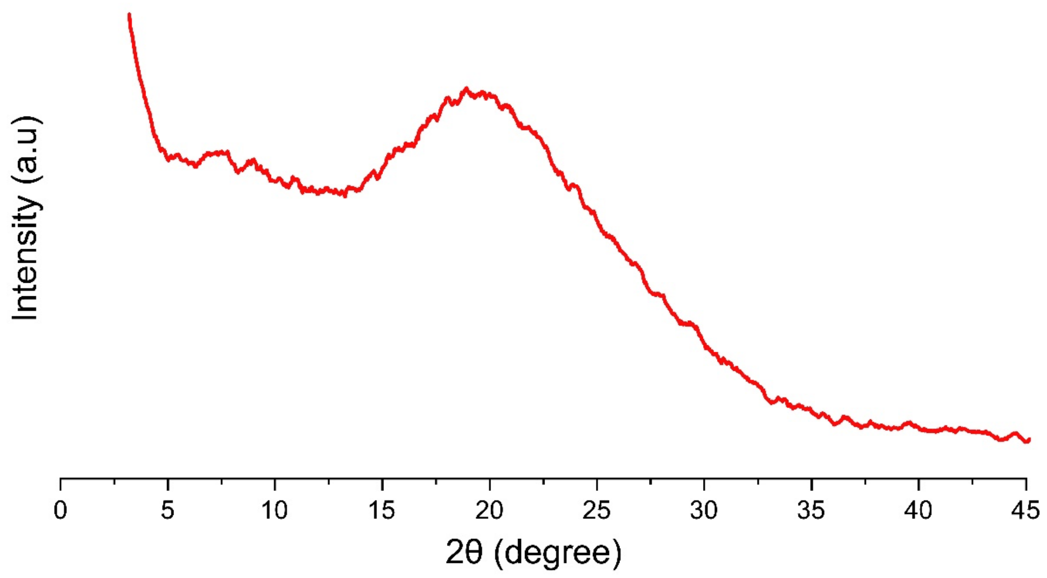

Figure S7: Experimental PXRD pattern of TTC-DTO.

PXRD measurements of TTC-PD revealed crystalline structure (Figure S5). The most intense diffraction peak at 2θ = 4.7° was assigned to the (100) plane, accompanied by additional reflections at 9.1°, 13.7°, 18.7°, and 25.8°, indexed to the (220), (120), (101), and (001) planes, respectively. Structural models based on eclipsed (AA) and staggered (AB) stacking were constructed using Forcite geometry optimization in Materials Studio (Figure S6). The experimental PXRD profile showed good agreement with the simulated AA-stacked model. Pawley refinement afforded optimized unit-cell parameters with satisfactory agreement factors (R_{wp} = 4.83%, R_p = 3.85%). Conversely, TTC-DTO displayed broad Bragg reflection, confirming its amorphous nature (Figure S7).

Section S-4: N_2 Sorption studies

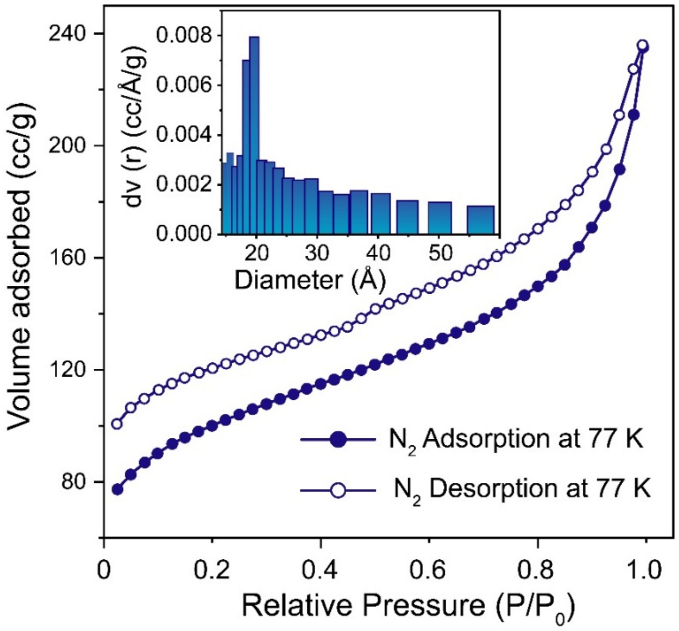

Figure S8: N_2 sorption isotherm of TTC-PD obtained at 77 K and the pore size distribution of TTC-PD obtained from the N_2 sorption isotherm using the nonlocal density functional theory (NLDFT) method.

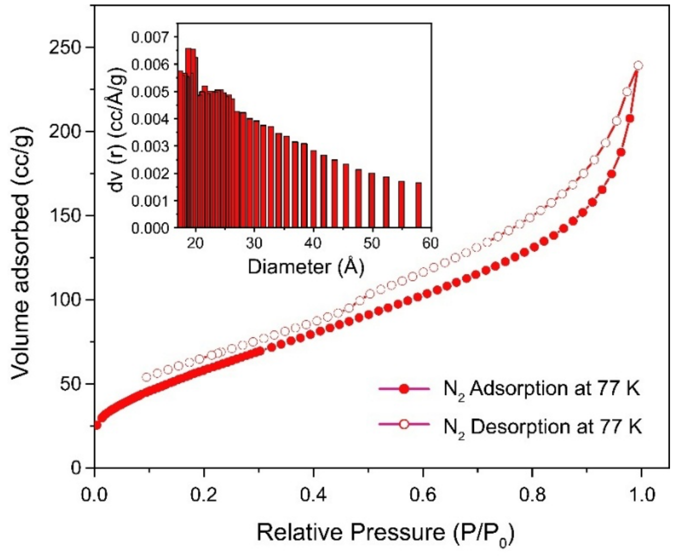

Figure S9: N_2 sorption isotherm of TTC-DTO obtained at 77 K and the pore size distribution of TTC-DTO obtained from the N_2 sorption isotherm using the NLDFT method.

The permanent porosity of TTC-PD and TTC-DTO was evaluated using N_2 sorption isotherms measured at 77 K (Figure S8-9). A type-II reversible absorption isotherm was observed in both cases. Brauner-Emmet-Teller (BET) surface areas of 336 m^2g^{-1} and 224 m^2g^{-1} were calculated for TTC-PD and TTC-DTO respectively. Pore size distributions calculated using NLDFT were centered around 20 Å for TTC-PD and 19 Å for TTC-DTO (Figure S8 & S9).

Section S-5: FT-IR, and ^{13}C CP-MAS solid-state NMR spectra analysis

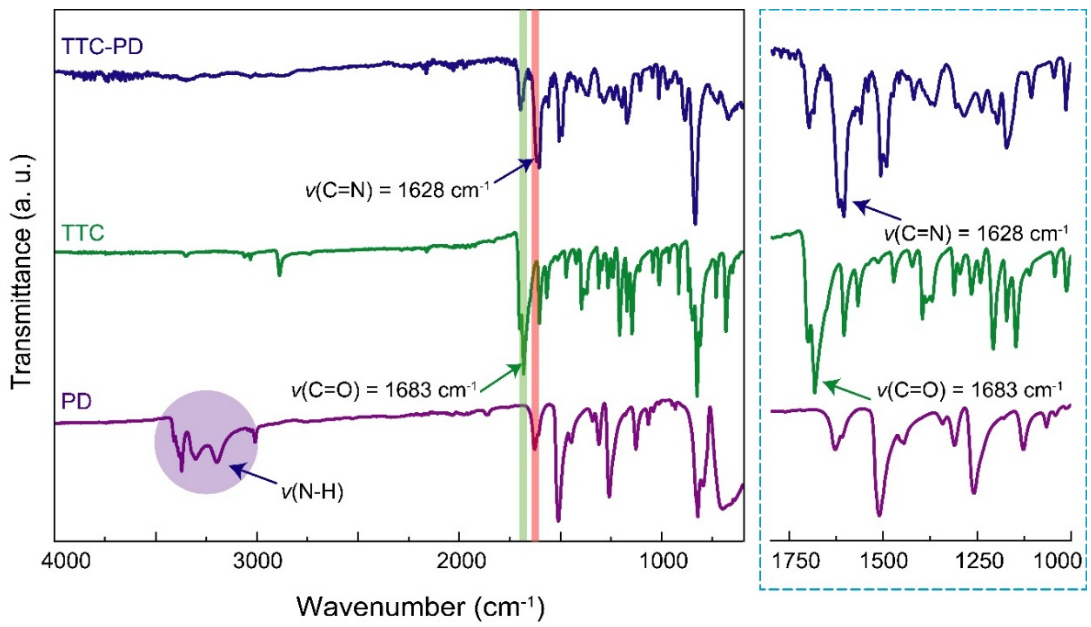

Figure S10: A comparison of the FT-IR spectra of TTC-PD with its corresponding starting materials, TTC and PD.

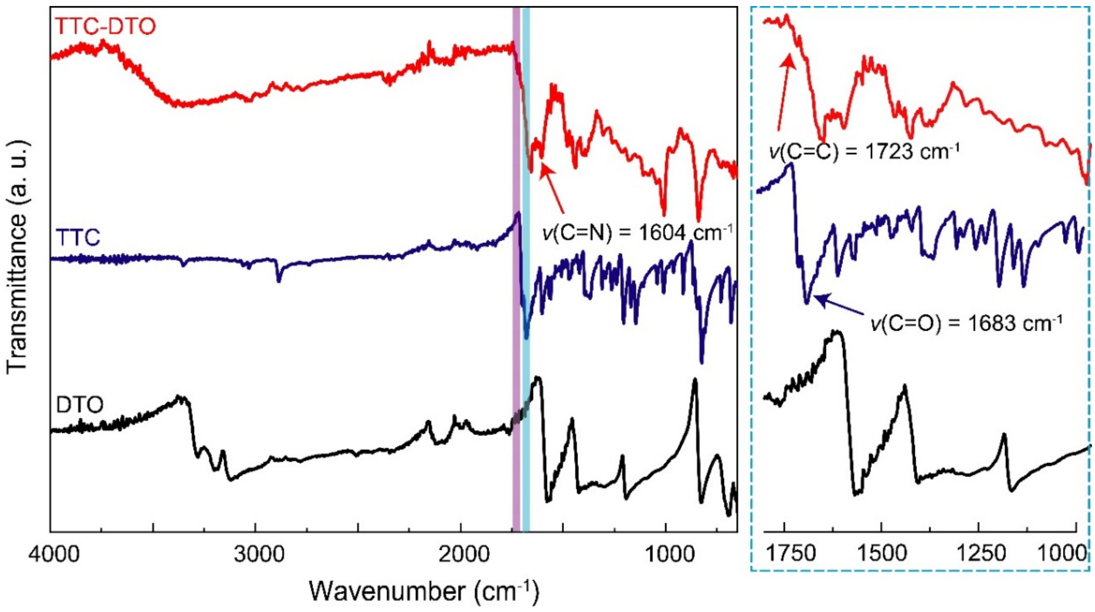

Figure S11: A comparison of the FT-IR spectra of TTC-DTO with its corresponding starting materials, TTC and DTO.

Consumption of the monomers (TTC and PD) and formation of the imine linkage in TTC-PD were established using Fourier transform infrared (FT-IR) spectroscopy (Figure S10 and S11). The characteristic carbonyl stretching band of TTC at 1683 cm^{-1} and the amine stretching bands of PD at 3340 cm^{-1} were absent in TTC-PD, while a band corresponding to the newly formed C=N bond appeared at 1628 cm^{-1} (Figure S10). Similarly, for TTC-DTO, the disappearance of the carbonyl and amine vibrational modes associated with the monomers (TTC and DTO) was observed (Figure S11). New vibrational bands attributed to the heterocyclic C=C and C=N bonds in TTC-DTO were observed at 1723 cm^{-1} and 1604 cm^{-1}, respectively, confirming the formation of the TzTz linkage.

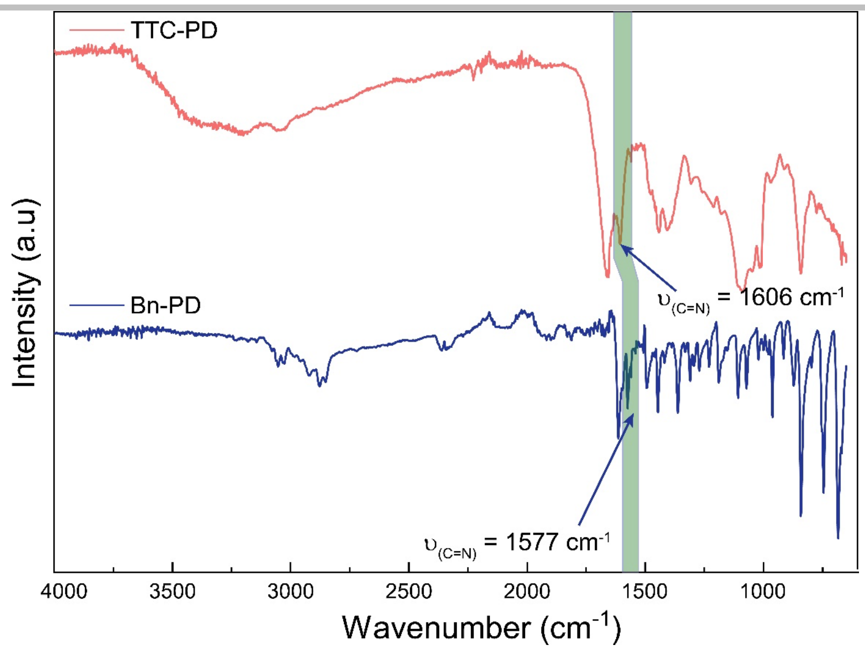

Figure S12: A comparison of FT-IR spectra of Bn-Pd with TTC-PD.

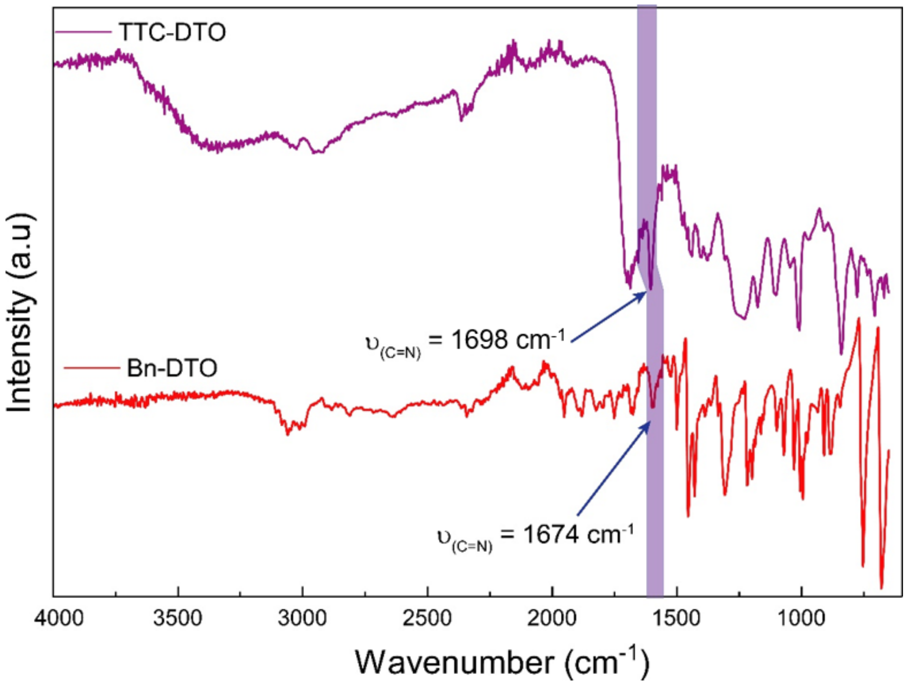

Figure S13: A comparison of the FT-IR spectra of Bn-DTO with TTC-DTO.

Comparison of the FT-IR spectra of TTC-PD and TTC-DTO with those of their corresponding model compounds revealed similar spectral features, with characteristic peaks slightly shifted to the higher wavenumber region for framework materials due to extended conjugation.

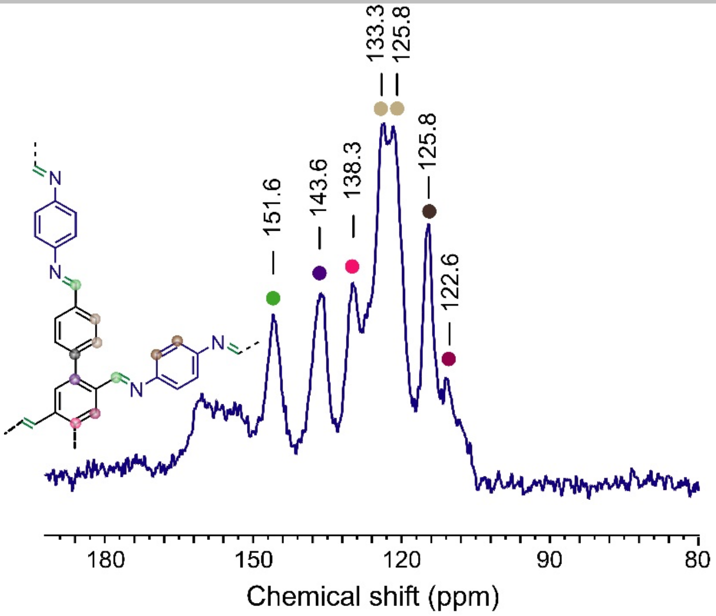

Figure S14: ^{13}C CP-MAS ssNMR spectra of TTC-PD (scan rate: 12500; number of scans: 4000).

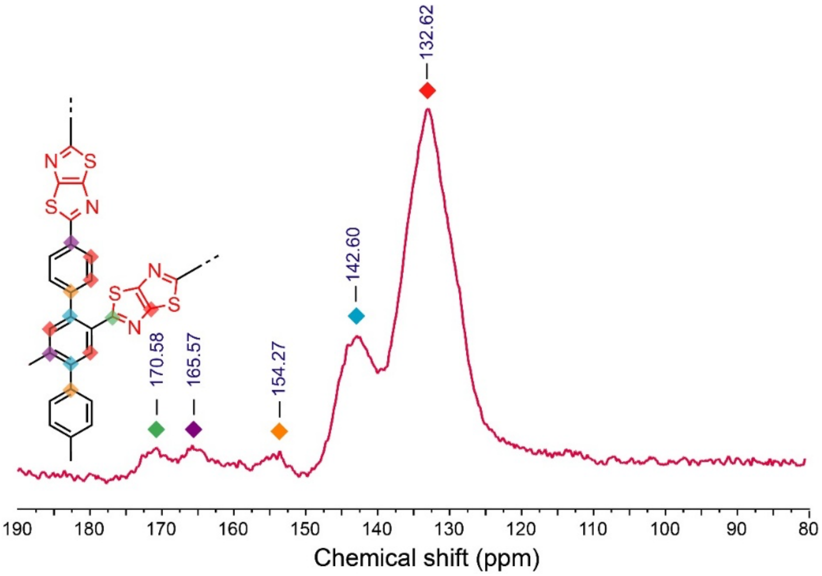

Figure S15: ^{13}C CP-MAS ssNMR spectra of TTC-DTO (scan rate: 12500; number of scans: 4000).

Further confirmation of the chemical composition of TTC-PD and TTC-DTO was obtained from their ^{13}C cross-polarization magic-angle spinning (CP-MAS) NMR spectra (Figure S14 and S15). For TTC-PD, the peaks corresponding to aromatic carbons appeared between 125 and 143 ppm (Figure S14). The signal corresponding to the imine carbon appeared at ~151.6 ppm. For TTC-DTO, peaks for aromatic carbons were distributed between 110 and 150 ppm (Figure S15). A higher chemical shift was observed for the carbons associated with the (bi)-heterocyclic TzTz ring, with peaks corresponding to the carbons of the S-C=N and C=C bonds appearing at ~170 ppm and 154 ppm, respectively (Figure S15).

Section S-6: Thermogravimetric analysis (TGA)

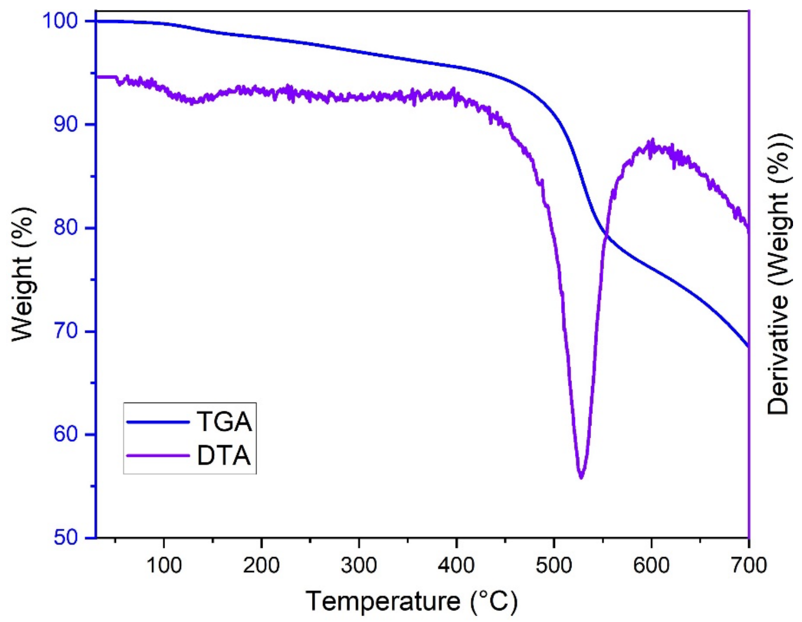

Figure S16: TGA and differential thermal analysis (DTA) curves of TTC-PD.

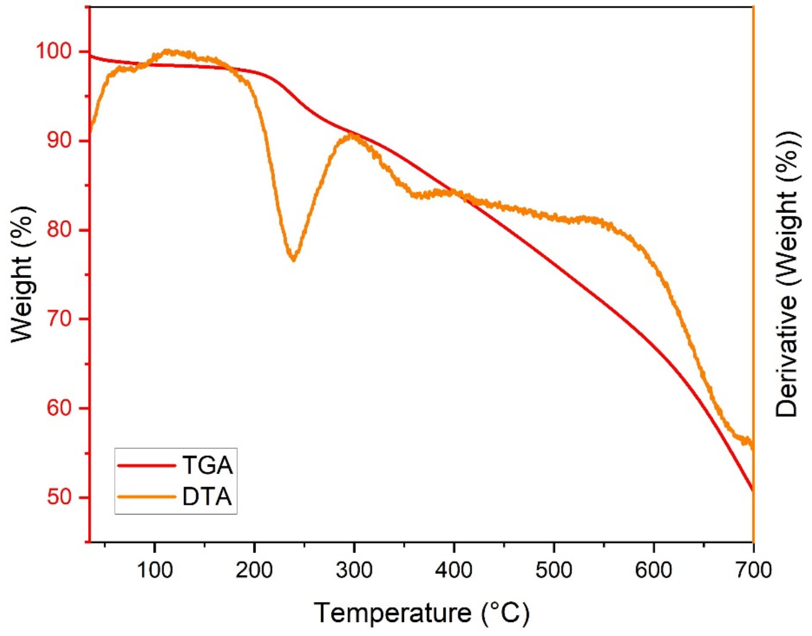

Figure S17: TGA and DTA curves of TTC-DTO.

Thermogravimetric analysis (TGA) indicated that TTC-PD and TTC-DTO were thermally stable up to ~400 ^{o}C and ~230 ^{o}C, respectively.

Section S-7: Electron microscopy imaging

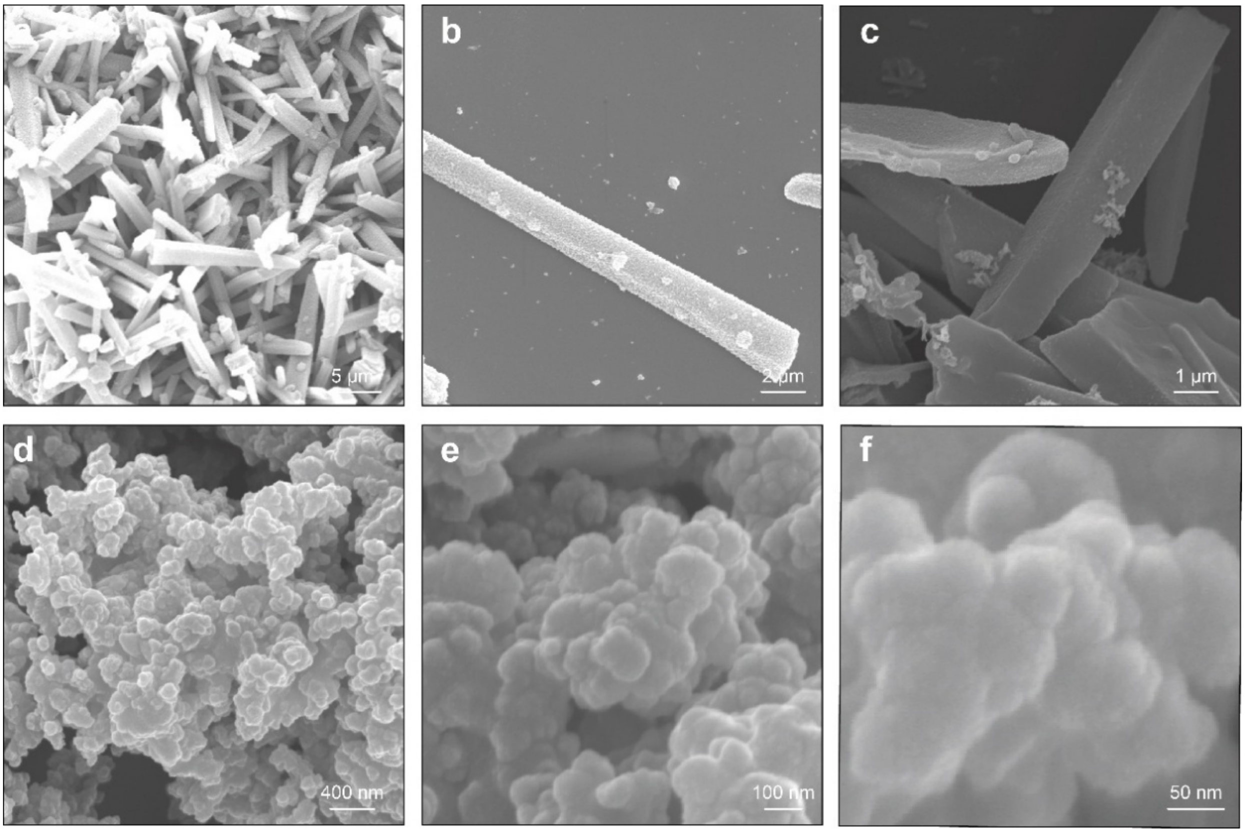

Figure S18: SEM images of (a-c) TTC-PD and (d-f) TTC-DTO. TTC-PD has a uniform rod-shaped morphology, while TTC-DTO has a globular morphology.

Electron microscopy imaging was performed to visualize the morphologies and structural features of TTC-PD and TTC-DTO (Figure S18 and S19). Both scanning electron microscopy (SEM) and high-resolution transmission electron microscopy (HRTEM) images of TTC-PD reveal a uniform rod-like morphology, with lengths in the range of ~15–20 μm (Figure S19 a-c). High-resolution TEM images further exhibit well-defined lattice fringes, indicative of ordered layered stacking within the framework (Figure S19 d-f). The corresponding selected area electron diffraction (SAED) pattern displays diffuse concentric rings, confirming the nature of the material with randomly oriented crystalline domains (Figure S19 (inset of (f)). On the other hand, TTC-DTO exhibits a uniform globular morphology composed of small spherical particulates with an average size of approximately 50 nm (Figure S19 g-i). Lattice fringes were not observed, and the corresponding SAED pattern displayed a diffused halo (Figure S19 (inset of (i)), consistent with amorphous materials.

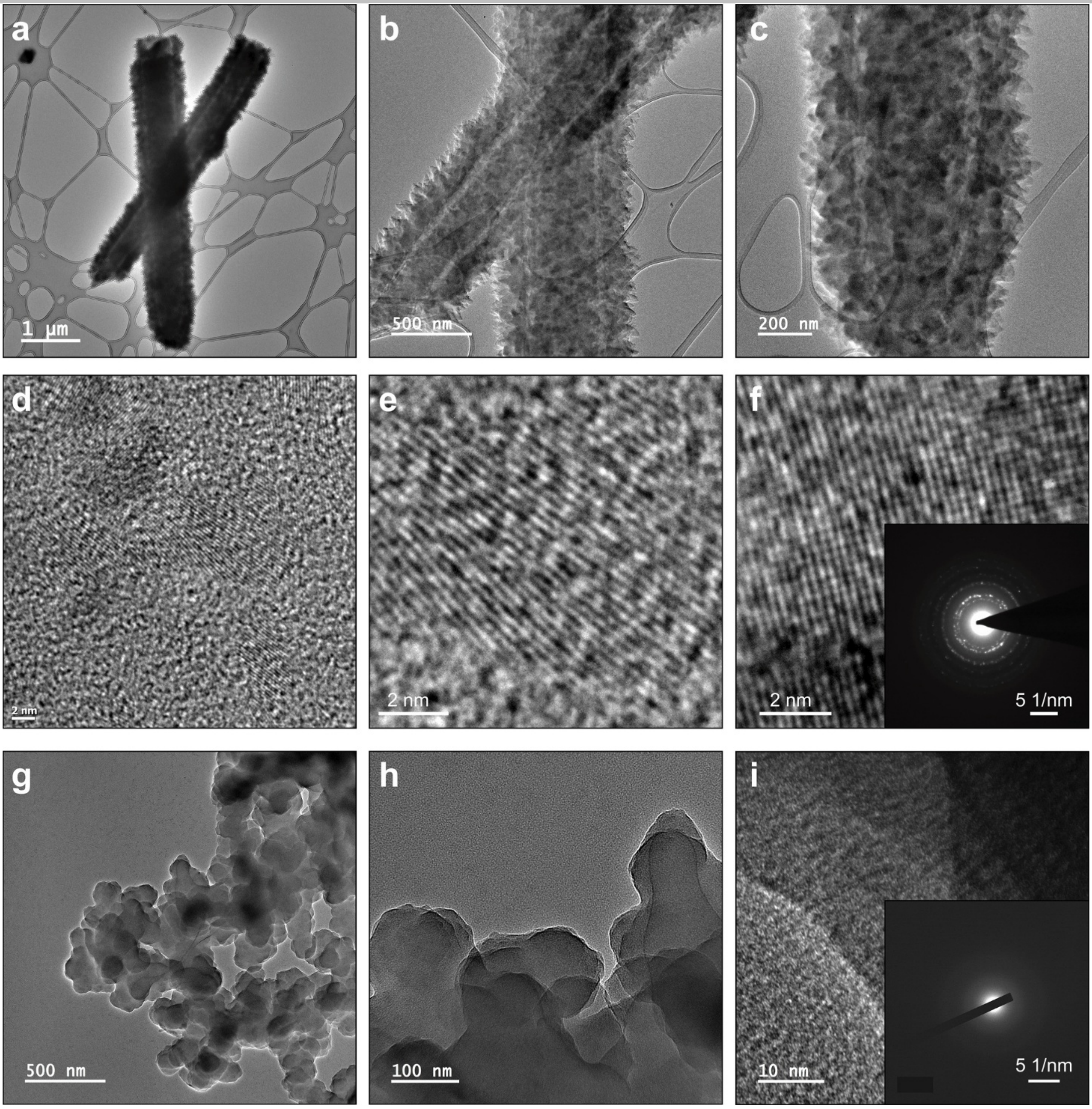

Figure S19: a-f) TEM images of TTC-PD, showing rod-like morphology and well-resolved lattice fringes in (d-f); inset of (f) shows the corresponding SAED pattern exhibiting diffuse concentric rings indicating a crystalline structure with randomly oriented crystalline domains; g-i) TEM images of TTC-DTO showing globular morphology composed of spherical particulates with no clearly resolved lattice fringes; inset of (i) shows the corresponding SAED pattern exhibiting a diffuse halo, indicative of an amorphous structure.

Section S-8: Theoretical Band-Structure Analysis

Computational Details

The electronic structure calculations were performed within the framework of Density Functional Theory (DFT) using the Vienna Ab-initio Simulation Package (VASP)[62-63]. The Generalized Gradient Approximation (GGA) with the Perdew-Burke-Ernzerhof (PBE) functional.[64-65] was employed for the exchange-correlation potential. The electronic wavefunctions were expanded using a plane-wave basis set with a kinetic energy cutoff of 520 eV.

Structural relaxation was performed until the total energy converged to within 10^{-6} eV. A force convergence threshold of 10^{-3} eV/Å per atom was applied for structural relaxation. For Brillouin-Zone (BZ) integration (Figure S19), Γ-centered k-point grids of 7 × 3 × 11 and 5 × 2 × 9 were adopted for the TTC-PD and TTC-DTO multilayer structures, respectively.

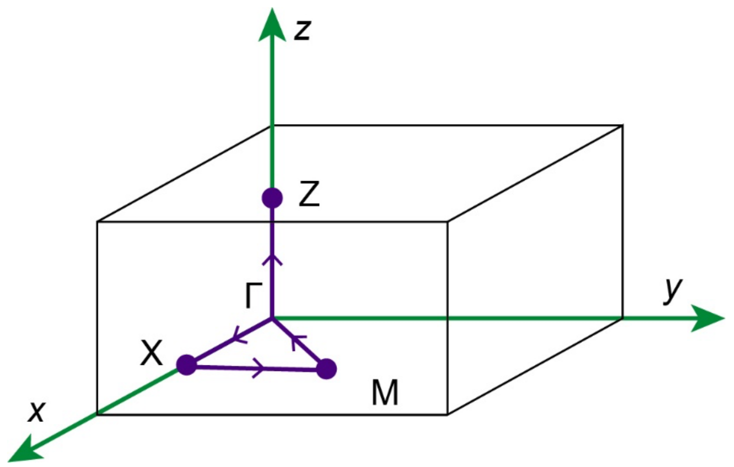

Figure S20: Schematics of the adapted Brillouin-Zone (BZ) with the mentioned sign conventions.

Electronic Properties

The electronic band structures were calculated along the k-path direction *Γ (0, 0, 0) → X (0.5, 0, 0) →M (0.5, 0.5, 0) → Γ (0, 0, 0) →Z (0, 0, 0.5)*. Both materials exhibit a direct band gap, with calculated values of 1.21 eV for TTC-PD and 1.31 eV for TTC-DTO.

For TTC-PD, the band edges display significant anisotropy. Flat-band features are observed in both the conduction and valence bands along the out-of-plane direction (*Γ → Z*), indicating limited charge carrier transport in the out-of-plane direction. In contrast, the valence band is more dispersed in the in-plane directions (*Γ → X → M → Γ*). This dispersion implies a lighter hole effective mass and, consequently, higher hole mobility within the plane compared to the out-of-plane direction. Furthermore, the in-plane curvature of the conduction band is greater than that of the valence band, suggesting that the electron effective mass is lighter than the hole effective mass, which would result in superior in-plane electron mobility.

In the case of TTC-DTO, the valence band dispersion is notably more anisotropic in the in-plane directions (*M→Γ* or *M→X*) compared to the conduction band. This anisotropy gives rise to a direction-dependent hole effective mass, favouring higher charge carrier transport along the *M→Γ* direction in the valence band. The calculated effective masses for both samples along the principal directions are summarized in Table S1.

Effective Masses

The effective mass tensor of holes (m_h^*) and electrons (m_e^*) was calculated with the following equation:

$$\left(\frac{1}{m^*}\right)_{ij} = \frac{1}{\hbar^2}\left(\frac{\partial^2 E_n(k^{\rightarrow})}{\partial k_i k_j}\right) (i, j = x, y, z) \qquad \ldots Eq.\ (i)$$

Where *x, y, z* are the directions in k-space and $E_n(\vec{k})$ is the dispersion relation of the nth electronic band. In the following table, we have tabulated the calculated effective mass of holes and electrons along different directions from the band structures of the samples using *vaspkit*[6] by fitting a third-order polynomial.

Based on the DFT calculated electron and hole effective masses along all symmetry allowed *k*-paths (Table S1), we observe that both TTC-PD and TTC-DTO exhibit strong anisotropy, with significantly lighter carrier masses along the Γ→X / Γ→M (TTC-PD) and M→Γ (TTC-DTO) directions compared to the other k-path directions. Since charge transport in these π-stacked frameworks is known to be dominated by in-plane delocalization, the relevant transport mass must be taken from these lowest-mass in-plane directions. Therefore, for each smaterial, we compute the in-plane reduced carrier mass (μ*) using the corresponding DFT electron and hole masses:

$$\mu = \frac{m_e^* m_h^*}{m_e^* + m_h^*} \qquad \ldots Eq.(ii)$$

SUPPORTING INFORMATION

Table S1: Calculated effective mass of holes and electrons calculated vasp-kit obtained by fitting a third-order polynomial.

**Material**	**K-path Direction**	**Electron (in m_0) (Prec.)**	**Hole (in m_0) (Prec.)**
**TTC-PD**	Γ →X	0.385 (1.5E-04)	-1.368 (5.0E-05)
	Γ →M	0.385 (4.2E-05)	-1.144 (1.4E-05)
	Γ →Z	6.664 (3.6E-08)	-4.079 (1.6E-08)
**TTC-DTO**	M →X	1.158 (3.7E-06)	-3.492 (2.2E-06)
	M→ Γ	1.010 (2.5E-06)	-0.601 (7.4E-06)
	M →Z	1.686 (1.1E-06)	-0.680 (1.1E-06)

Applying this relation to the in-plane electron and hole masses yields $\mu^* = 0.294m_0$ for TTC-PD and $\mu^* =0.623m_0$ for TTC-DTO. These values represent the physically meaningful, transport-relevant reduced masses because they capture the effective curvature of the bands along the directions that contribute most strongly to THz-frequency conduction. Accordingly, these reduced masses are used as the default effective mass input in all Drude-Smith fits reported in the main text.

Table S2: Effective reduced mass of carriers in TTC-PD and TTC-DTO from theoretically calculated band structures used for primary fitting.

**Material**	**In-plane μ^* (m_0)**
TTC-PD	0.294
TTC-DTO	0.623

Section S-9: THz spectroscopy and optical property analysis

Terahertz (THz) measurements and data analysis:

Frequency domain THz spectroscopy:

FDTS spectra of Bn-PD, Bn-DTO, TTC-PD, and TTC-DTO were recorded in a Bruker Vertex 70V FTIR setup, which operates under vacuum to avoid moisture. An Hg-arc lamp was used as the continuous band light source instead of a pulsed laser to achieve spectral data collection over the extended frequency region. A pellet of the pure sample was prepared for all measurements. The optoelectronic properties were determined using the following methodology: First, the absorption coefficients of the samples in the THz region were calculated using their transmittance spectra.

The optoelectronic properties reported from the frequency domain terahertz spectroscopic data were calculated using the Kramers-Kronig Transformations (KKT), which connects the real and imaginary parts of linear complex optical functions.[43,44,48] The absorption coefficient $\alpha(v)$ is calculated from transmittance $T(v)$ and sample thickness d as:

$$\alpha(\nu) = -\frac{lnT(\nu)}{d} \qquad \text{...Eq. (iii)}$$

The imaginary part of the refractive index, also known as the extinction coefficient, $k(v)$, is then calculated from the absorption coefficient given by the relation,

$$\kappa(\nu) = -\frac{\alpha(\nu)c}{4\pi\nu} \qquad \text{...Eq (iv)}$$

Where *c* is the speed of light in vacuum in *cm/s*.

The real part of the complex refractive index, *n*(v), is obtained by using KKT applied to k(v):

$$n(\nu) = \frac{2}{\pi} P \int_0^\infty \frac{\nu\prime\kappa(\nu\prime)}{\nu\prime^2-\nu^2} + n(\infty) \qquad \text{...Eq (v)}$$

Here, P is the Cauchy principal value and $n(\infty)$ is the refractive index of the material at infinite wavenumber. In practice, the integral is calculated up to the highest measured wavenumber in the absorbance spectrum.[43,48] The average refractive index value at the highest measurable range in the mid-IR region is used for (∞). The $n(\infty)$value was obtained using the KKT of reflectance measurements in the MIR region from OPUS software. All other calculations are done using MATLAB.

Further, the complex dielectric function and the optical conductivity of the material are determined from the components of the complex refractive index. The real and imaginary parts of the dielectric function $\varepsilon = \varepsilon' + i\varepsilon''$ are calculated as:

$$\varepsilon'(\nu) = n^2(\nu) - \kappa^2(\nu) \qquad \text{... Eq. (vi)}$$

$$\varepsilon''(\nu) = 2n(\nu)\kappa(\nu) \qquad \text{...Eq. (vii)}$$

The real part of the optical conductivity is given by

$$\sigma_r(\nu) = \omega\varepsilon_0\varepsilon'(\nu) \qquad \text{...Eq. (viii)}$$

$$\sigma_i(\nu) = \omega\varepsilon_0\,[\varepsilon_\alpha(\nu) - n^2(\nu) + \kappa^2(\nu)] \qquad \text{... Eq. (ix)}$$

Where, $\omega = 2\pi v$, ε_0 is the vacuum permittivity in *F/cm*, and ε_α is the static dielectric constant, assumed to be 1.

Table S3: Average thickness of Pallets prepared. The same pallets were used for all THz measurements.

Sample Name	Thickness of pellets (in mm)
Bn-PD	0.570
Bn-DTO	0.876
TTC-PD	0.590
TTC-DTO	0.950

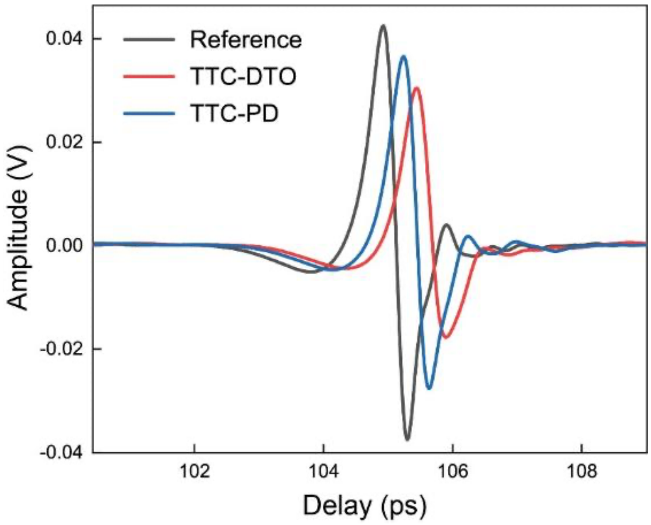

Figure S21: THz time-domain Pulse of reference, TTC-PD, and TTC-DTO.

Time domain THz spectroscopy (TDTS):

Transmission-mode terahertz time-domain spectroscopy (TDTS) measurements were carried out in the frequency range of 0.5–1.6 THz. The setup utilized a mode-locked Ti:sapphire femtosecond laser (Synergy model) that generated 10-fs pulses centered at 800 nm with a repetition rate of 78 MHz.

These optical pulses illuminated a low-temperature-grown GaAs (LT-GaAs) photoconductive antenna (BATOP GmbH) to generate sub-picosecond THz radiation. The emitted THz pulses were detected via electro-optic sampling in a <110> cut ZnTe crystal. The THz beam was transmitted through sample pellets under ambient conditions (Figure S20). Reference and sample THz transmission waveforms were recorded in the time domain, and their complex spectra were obtained through fast Fourier transformation (FFT).

Further, refractive index (*n(ω)*), extinction coefficient (*k(ω)*), dielectric constants (*ε'(ω)* and *ε''(ω)*) as a function of frequency were retrieved by the formulae:

$n(\omega) = 1 - \frac{c\phi(\omega)}{\omega d}$, ...*Eq. (X)*

$k(\omega) = ln\left[\frac{4n}{(n+1)^2\rho(\omega)}\right]\frac{c}{\omega d}$, *Eq. (Xi)*

$\varepsilon'(\omega) = n(\omega)^2 - k(\omega)^2$ *Eq. (Xii)*

$\varepsilon''(\omega) = 2n(\omega)k(\omega)$. ...*Eq. (Xiii)*

Here, $\rho(\omega)$ denotes the amplitude ratio of the electric fields between the reference and sample THz pulses, $\phi(\omega)$ represents the corresponding phase difference, and d is the thickness of the pellet. Additionally, the frequency-dependent real ($\sigma'(\omega)$) and imaginary ($\sigma''(\omega)$) parts of the conductivity of the samples were found using the formula:

$$\sigma'(\omega) = \omega\varepsilon_0\varepsilon''(\omega) \qquad \text{...Eq. (Xiv)}$$

$$\sigma''(\omega) = \omega\varepsilon_0(\varepsilon_\infty - \varepsilon'(\omega)) \qquad \text{...Eq. (Xv)}$$

Further, the THz-conductivity was modelled using the Drude-Smith function detailed later (*vide infra*)

ATR Measurement:

The framework material TTC-PD and TTC-DTO have higher absorption in the extended region 1-20 THz. So, the measurement using the same pellet that was used in the lower region is difficult. We employ the ATR method to check the absorption feature in the extended region. For this, we used ATR accessories and kept the system under vacuum for moisture-free free and other parameters were kept constant. From the experimental spectra, it was clearly seen that both TTC-PD and TTC-DTO have many absorption features in the extended region.

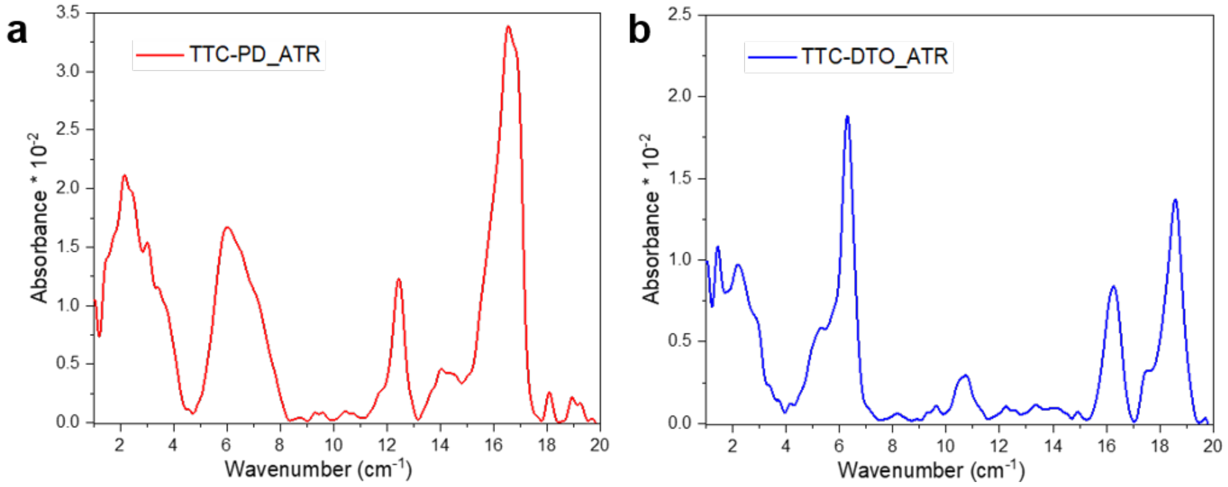

Figure S22: The frequency domain ATR-IR spectrum of a) TTC-PD and b) TTC-DTO showing different absorption features in the extended region from 1-20 THz region.

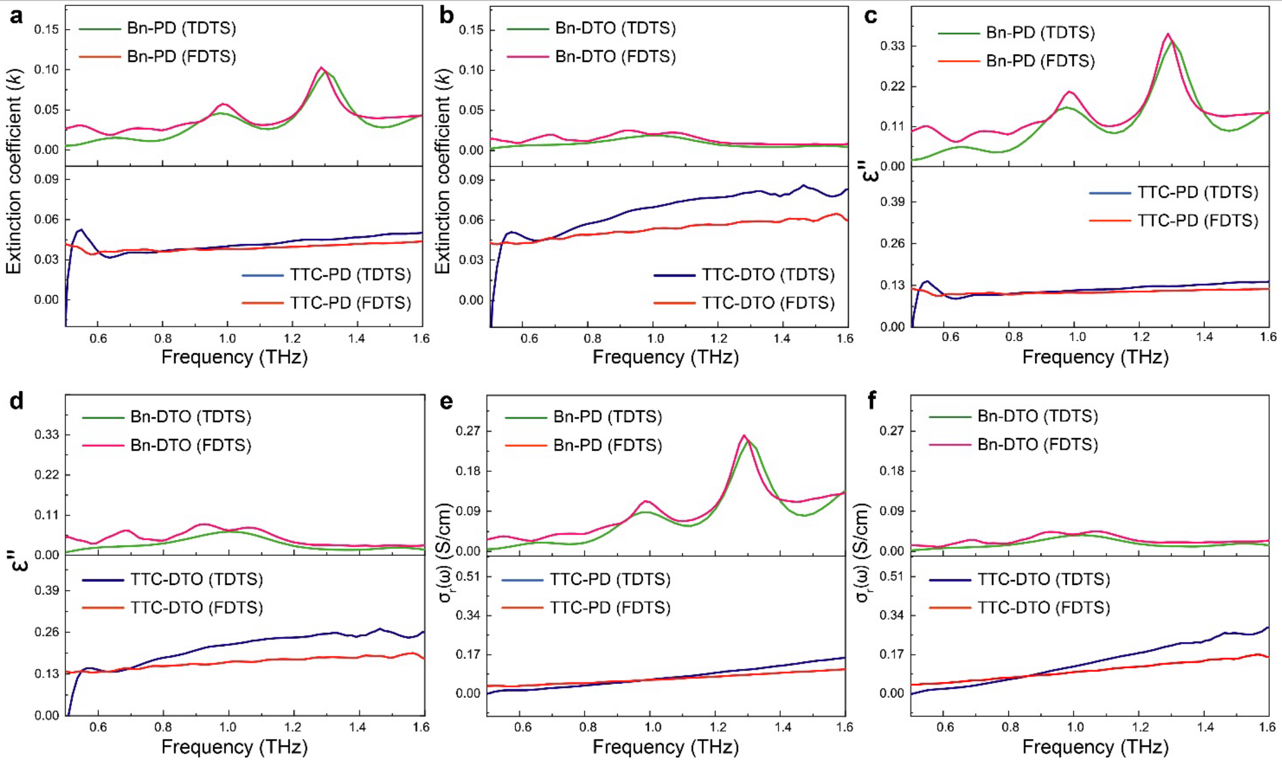

Figure S23. Different optoelectronic properties in the range of 0.5-1.6 THz region: a, b) Comparison of the extinction coefficient (*k*) of TTC-PD and TTC-DTO with their respective molecular analogues Bn-PD and Bn-DTO; The *k* of TTC-PD and TTC-DTO ranges from 0.039 to 0.042 and 0.040 to 0.062, respectively. TTC-DTO has a slightly higher *k*, suggesting greater light absorption. For Bn-PD (0.03-0.1) and Bn-DTO (0.01-0.007), variation occurs due to different absorption peaks. c, d) Comparison of the imaginary part of the dielectric function (ε'') of TTC-PD and TTC-DTO with their respective molecular analogues Bn-PD and Bn-DTO. The imaginary part of the dielectric function (ε'') of TTC-PD and TTC-DTO ranges from 0.13 to 0.19, remaining almost constant. In the cases of Bn-PD (0.01-0.36) and Bn-DTO (0.05-0.02), variation is observed due to the presence of different absorption peaks. The molecular analogues Bn-PD and Bn-DTO have consistently lower ε'' value than TTC-PD and TTC-DTO. e, f) Comparison of real part of optical conductivity $\sigma_r(\omega)$ of TTC-PD and TTC-DTO with their respective molecular analogues Bn-PD and Bn-DTO. The $\sigma_r(\omega)$ value of TTC-PD and TTC-DTO in the range of 0.5-1.6 THz varies from 0.03-0.10 and 0.04-0.16 S/cm, respectively. This variation in Bn-PD and Bn-DTO in the same region is from 0.03 to 0.25 and 0.02 to 0.05 S/cm, respectively.

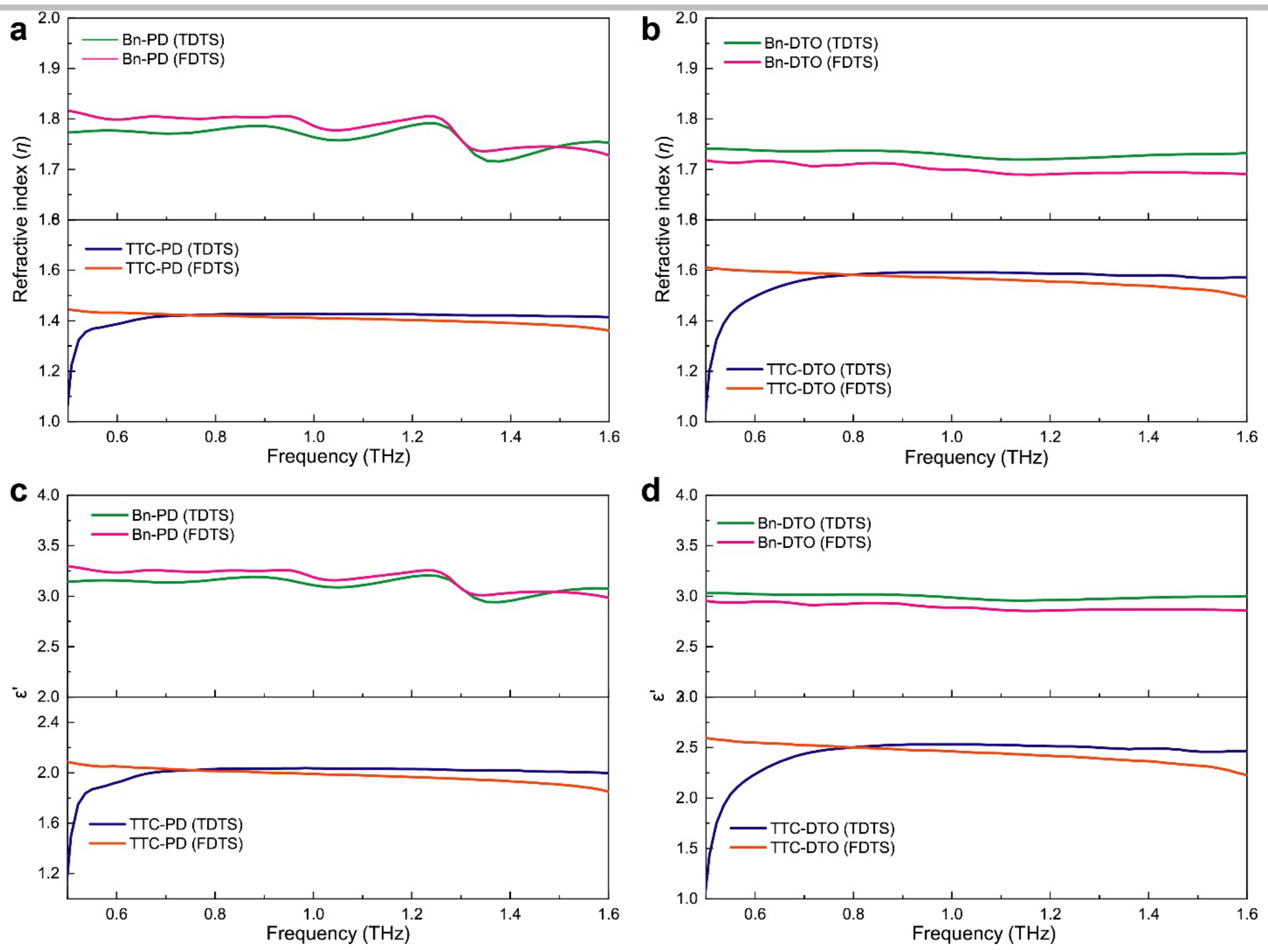

Figure S24: Different optoelectronic properties in the range of 0.5-1.6 THz region: a, b) Comparison of the real part of refractive index (η) of TTC-PD and TTC-DTO with their respective molecular analogues Bn-PD and Bn-DTO; The variation of η for TTC-PD and TTC-DTO is from 1.45 to 1.36 and 1.60 to 1.50, respectively, in the 0.5 to 1.6 THz. This reveals that the TTC-DTO has consistently higher η than TTC-PD throughout the spectral range. The variation of η for Bn-PD and Bn-DTO is from 1.80 to 1.70 and 1.71 to 1.69, respectively, in the range 0.5-1.6 THz. c, d) Comparison of the real part of the dielectric function (ε') of TTC-PD and TTC-DTO with their respective molecular analogues Bn-PD and Bn-DTO. The ε' of TTC-PD and TTC-DTO varies from 2.07 to 1.87 and 2.58 to 2.26, with consistently higher ε' for TTC-DTO compared to TTC-PD. The variation of ε' of Bn-PD and Bn-DTO is from 3.22 to 3.00 and 2.94 to 2.86. The molecular analogues Bn-PD and Bn-DTO have consistently higher ε' value than TTC-PD and TTC-DTO.

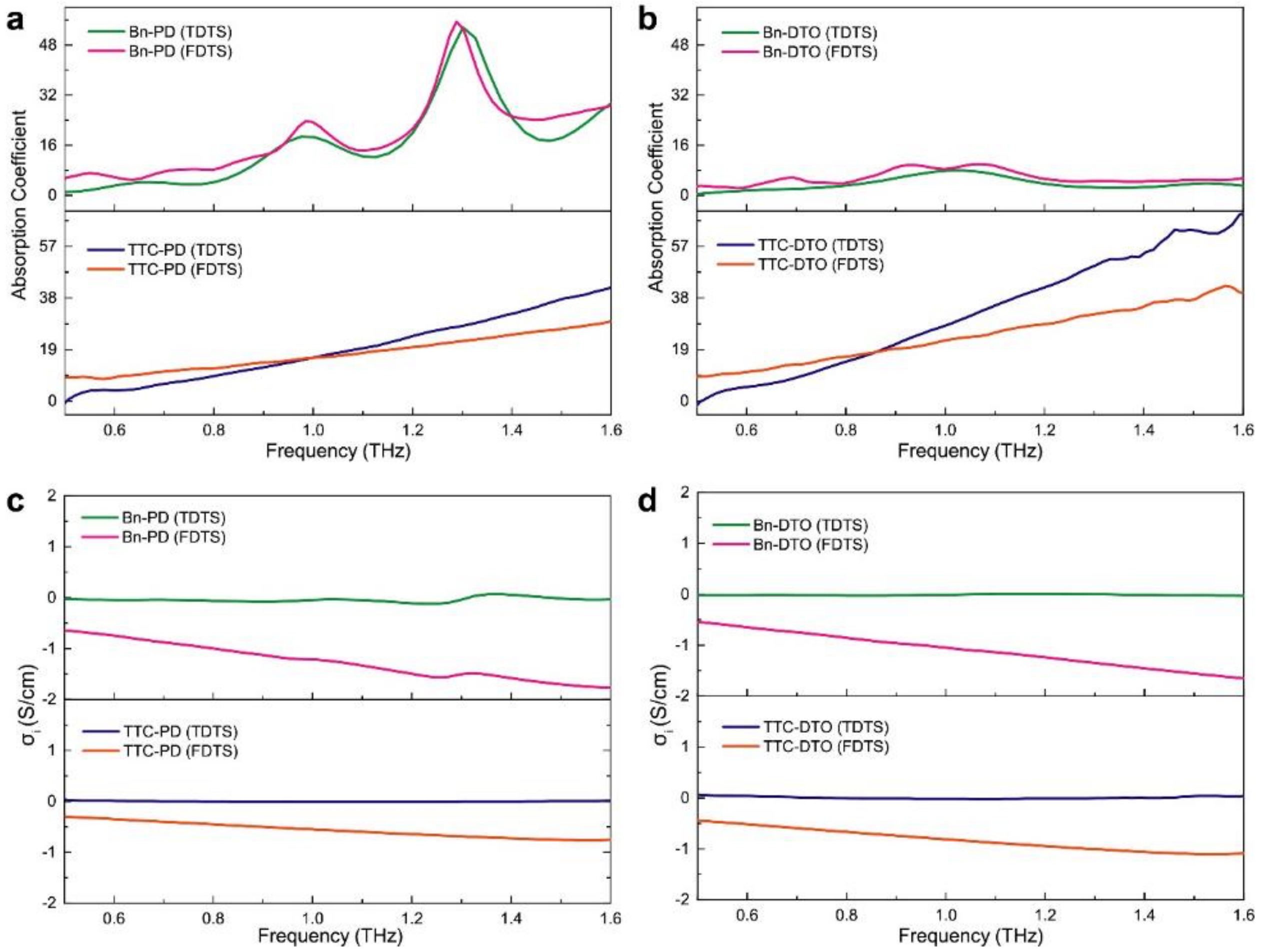

Figure S25: Different optoelectronic properties in the range of 0.5-1.6 THz region: a, b) Comparison of the absorption coefficient (*α*) of TTC-PD and TTC-DTO with their respective molecular analogues Bn-PD and Bn-DTO; The *α* value for TTC-PD and TTC-DTO varies from 8 to 29 and 8 to 41 $cm^{-1,}$ respectively. In case of Bn-PD, this variation is more (6 to 54 cm^{-1}) than Bn-DTO (3-10 cm^{-1}), respectively. The *α* of Bn-PD has a higher value than TTC-PD, and Bn-DTO has a lower *α* value than TTC-DTO. c, d) Comparison of the imaginary part of optical conductivity ($\sigma_i(\omega)$) of TTC-PD and TTC-DTO with their respective molecular analogues Bn-PD and Bn-DTO. The $\sigma_i(\omega)$ value of TTC-PD and TTC-DTO in the range of 0.5-1.6 THz varies from -0.31 to -0.77 and -0.44 to -1.10 S/cm, respectively. The consistently more negative $\sigma_i(\omega)$ for TTC-DTO suggests a stronger capacitive response compared to TTC-PD. This variation in Bn-PD and Bn-DTO in 0.5-1.6 THz is from -0.68 to -1.80 and -0.56 to -1.63 S/cm, respectively. The variation of $\sigma_i(\omega)$ is consistently higher for molecular analogues (Bn-PD and Bn-DTO) than for framework materials (TTC-PD and TTC-DTO).

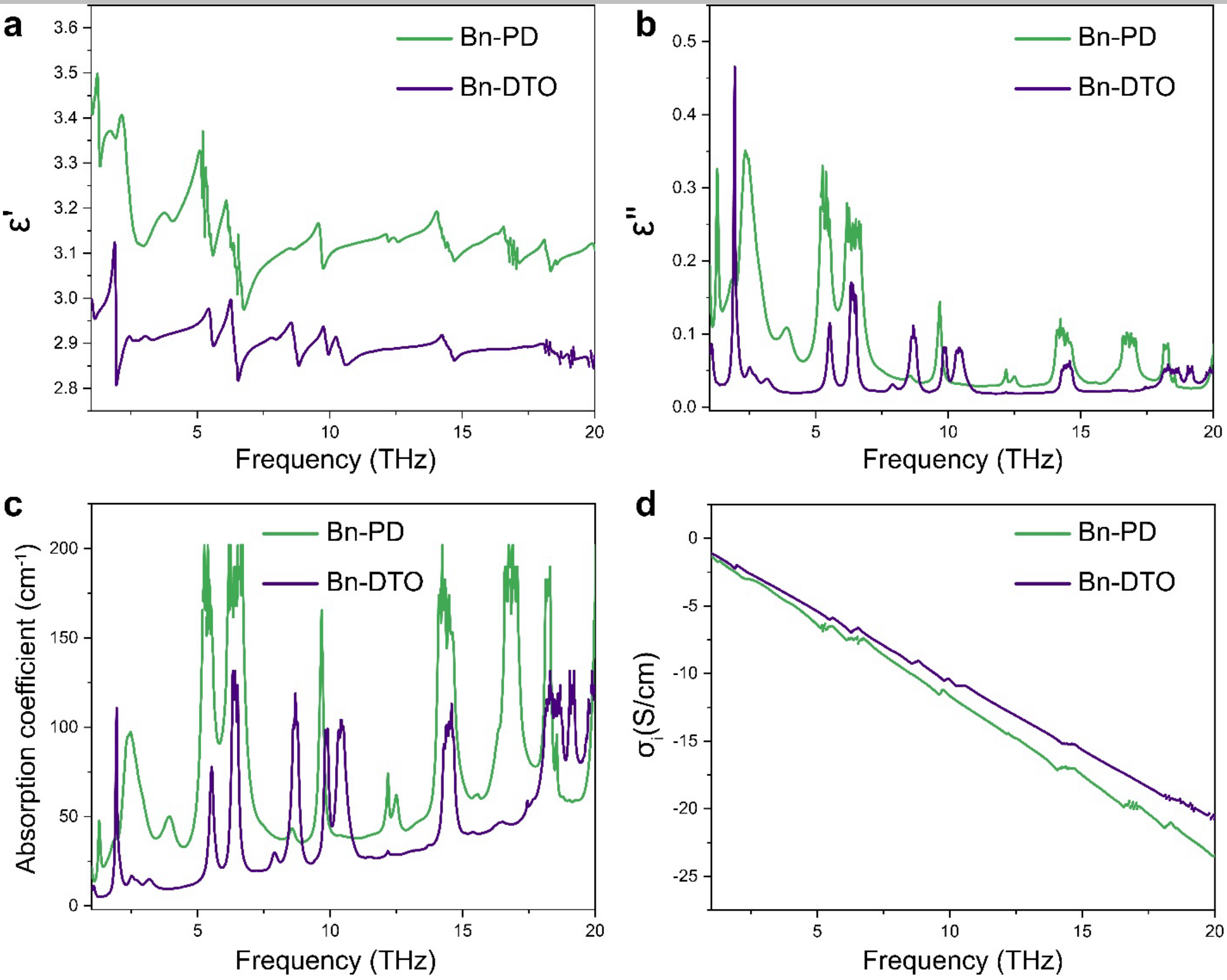

Figure S26: Different optoelectronic properties in the range of 1-20 THz region: a) The variation of the real part of the dielectric function (ε') of molecular analogues Bn-PD and Bn-DTO; The variation of ε' from 3.47 to 2.99 and 3.10 to 2.87, respectively. b) The variation of the imaginary part of the dielectric function (ε'') of molecular analogues Bn-PD and Bn-DTO; The variation of ε'' from 3.20 to 0.05 and 4.70 to 0.05, respectively c) The absorption coefficient (α) of molecular analogues Bn-PD and Bn-DTO; For Bn-PD (18-200 cm^{-1}) and Bn-DTO (10-130 cm^{-1}), variation occurs due to different absorption peaks. d) The imaginary part of optical conductivity ($\sigma_i(\omega)$) of molecular analogues Bn-PD and Bn-DTO. The variation $\sigma_i(\omega)$ in Bn-PD and Bn-DTO is -1.70 to -6.27 and -1.34 to -5.36 S/cm, respectively.

Table S4. A summary of the variation of optical parameters of various frameworks and molecular analogue samples in the 0.5 to 1.6 THz region

Samples	*η*	*ε'*	*k*	*ε"*	*α*(cm^{-1})	$\sigma_r(\omega)$ (S/cm)	$-\sigma_i(\omega)$ (S/cm)
**TTC-PD**	1.45–1.36	2.07–1.87	0.039–0.042	0.13–0.19	8–29	0.03–0.10	0.31–0.77
**TTC-DTO**	1.60–1.50	2.58–2.26	0.040–0.062	0.13–0.19	4–41	0.04–0.16	0.44–1.10
**Bn-PD**	1.80–1.70	3.22–3.00	0.03–0.1	0.01–0.36	6–54	0.03–0.25	1.22–1.73
**Bn-DTO**	1.71–1.69	2.94–2.86	0.01–0.007	0.05–0.02	3–10	0.02–0.05	0.56–1.63

Extended Analysis of THz Optical Constants and Dielectric Properties:

Both TTC-PD and TTC-DTO exhibit very low values of the extinction coefficient (*k*), ranging from 0.039 to 0.042 and 0.040 to 0.062, respectively, within the wavelength region of 0.5-1.6 THz (Figure S23a, b). The *k* is the direct measurement of how strongly a material absorbs light. A lower value of *k* for the material signifies their more transparent nature in the THz region.[54] Furthermore, as TTC-DTO has consistently higher variation of *k* compared to TTC-PD, it can be inferred that TTC-DTO interacts more with THz light. This value of *k* for Bn-PD (0.03-0.1) and Bn-DTO (0.01-0.007), respectively, in the same range (Figure 23a, b) and 0.018 to 0.096 and 0.005 to 0.133, respectively in the extended THz range (Figure 4b, main manuscript). Bn-PD has a higher *k* value than TTC-PD, indicating it absorbs more THz light, while Bn-DTO has a lower *k*, implying it absorbs less THz light than TTC-DTO.

The imaginary part of the dielectric function (*ε"*) of TTC-PD and TTC-DTO ranges from 0.13 to 0.19, remaining almost constant across the 0.5-1.6 THz wavelength range (Figure 23c, d). The *ε"* represents energy dissipation or absorption within the material caused by the applied electric field. It is directly related to the material's conductivity and absorption coefficient, which contribute to energy loss as heat. A small *ε"* indicates minimal absorption of THz radiation by the material. Most of the THz wave energy is not converted into other energy forms, like heat from molecular vibrations or free carrier absorption.[55] In the cases of Bn-PD (0.01-0.36) and Bn-DTO (0.05-0.02), variation is observed due to the presence of different absorption peaks. The molecular analogues Bn-PD and Bn-DTO have consistently lower *ε"* values than the framework materials TTC-PD and TTC-DTO. On the other hand, The absorption coefficient (*α*) is a crucial parameter in the THz range as it quantifies how strongly a material absorbs THz radiation at a specific frequency. It directly relates to the rate at which the intensity of the THz wave decreases as it propagates through the material. The *α* value for TTC-PD and TTC-DTO varies from 8 to 29 and 8 to 41 cm^{-1}, respectively, in 0.5 to 1.6 THz (Figure S26 a,b). TTC-PD, with the imine linkage, seems to preserve more of the THz signal, resulting in a lower value of *α*, possibly due to the more ordered or crystalline structure, which reduces phonon scattering. In the case of Bn-PD, this variation is more (6 to 54 cm^{-1}) than Bn-DTO (3-10 cm^{-1}) within the wavelength region of 0.5-1.6 THz (Figure S26 a,b). The *α* of Bn-PD has a higher value than TTC-PD, and Bn-DTO has a lower *α* value than TTC-DTO.

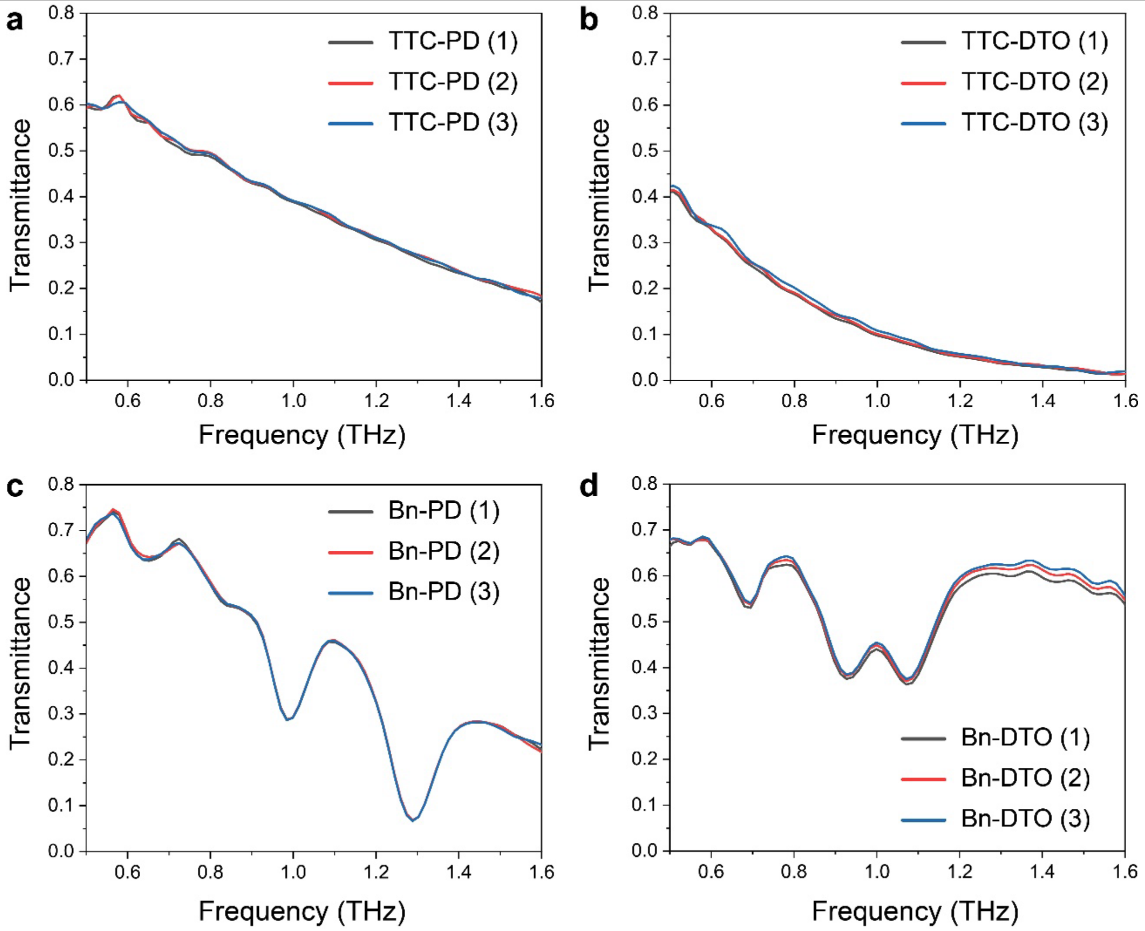

Figure S27: Transmittance spectra of a) TTC-PD, b) TTC-DTO, c) Bn-PD, and d) Bn-DTO over three repeated measurements in the FDTS mode, showing the reproducibility of the data. The numbers 1, 2, and 3 show the repeated measurements of each sample.

Quantitative agreement between TDTS and FDTS data:

The quantitative agreement between TDTS and FDTS measurements is demonstrated through correlation and Bland–Altman analyses of the transmittance spectra for both TTC-PD and TTC-DTO. The correlation plots exhibit excellent linearity, with R^2 = 0.995 for TTC-PD and 0.991 for TTC-DTO, indicating that both techniques capture nearly identical spectral features across the measured range. This is further supported by the low root-mean-square error (RMSE) values of 0.194 and 0.103 for TTC-PD and TTC-DTO, respectively. The Bland-Altman plots reveal that these deviations are predominantly systematic rather than random, with no significant frequency-dependent discrepancies. Together, these results demonstrate that the optical response obtained from FDTS is quantitatively consistent with TDTS measurements in the overlapping spectral region, thereby validating the reliability of the FDTS-derived optical constants and conductivity used for subsequent transport analysis.

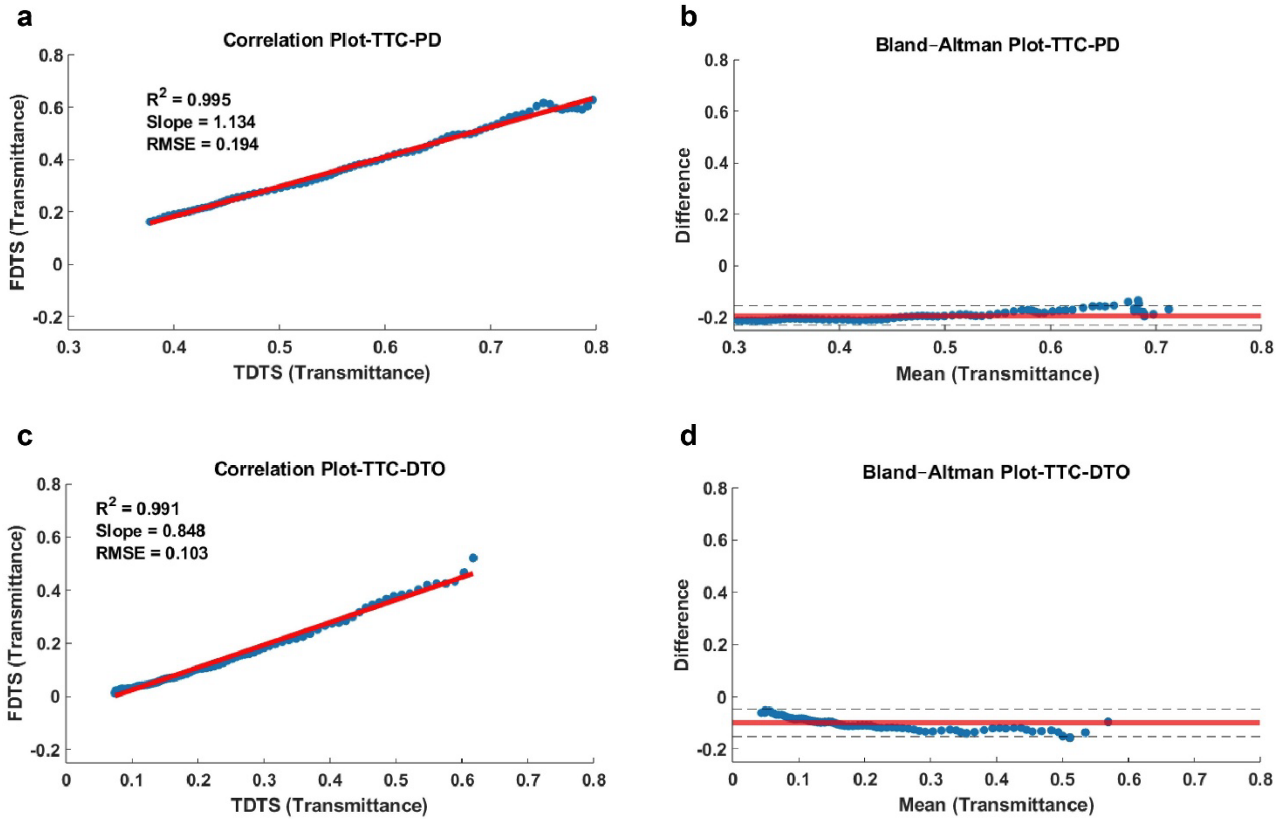

Figure S28: Quantitative comparison of FDTS and TDTS transmittance spectra. a, c) Correlation plots between FDTS and TDTS transmittance values, along with linear fits (red line), demonstrating strong linear agreement; b,d) Corresponding Bland-Altman plots showing the difference between FDTS and TDTS transmittance as a function of their mean, with the red solid line indicating the mean bias and dashed lines representing the limits of agreement.

Drude-Smith (DS) fitting of THz conductivity:

TTC-PD and TTC-DTO materials show strong suppression of the real part of the conductivity at low frequencies, together with a negative imaginary part-both signatures of charge-carrier localization. These spectral characteristics cannot be satisfactorily captured by the conventional Drude model alone. Instead, excellent agreement with the experimental data is achieved using the Drude-Smith (DS) formalism, an extension of the classical Drude theory that incorporates partial retention of carrier velocity (backscattering) during scattering events.

The Drude-Smith expression is given by:

$$\sigma(\omega) = \frac{\varepsilon_0 {\omega_p}^2 \tau}{(1-i\omega\tau)}\left(1 + \frac{c_1}{1-i\omega\tau}\right) \quad \text{...Eq. (xvi)}$$

Where ω_p is the plasma frequency, τ is the average momentum scattering time, ε_0 is the vacuum permittivity, and c_1 (typically $-1 \leq c_1 \leq 0$) is the velocity-persistence (backscattering) parameter. A value of $c_1 = 0$ recovers the standard Drude model (complete velocity randomization upon collision), whereas negative c_1 values indicate preferential backscattering, a common feature in disordered, nanostructured, or spatially confined systems.

For TTC-PD and TTC-DTO the values of c_1 were approximately -0.86 and -0.95, confirming dominant backscattering of charge carriers. Excellent agreement between the model and the experimental complex conductivity spectra of both TTC-PD and TTC-DTO obtained using the Drude-Smith expression is shown in Figure S24. The free-charge-carrier density (n) and mobility (μ_{DC}), were then obtained from DS model fitted parameters: ω_p and τ, using the formula

$$n = \frac{\varepsilon_0 m^* {\omega_p}^2}{e^2} \quad \text{...Eq. (xvii)}$$

and, $\mu_{DC} = \frac{e\tau}{m^*}$ ...*Eq. (xviii)*

This reflects localized transport dominated with nearly complete backscattering. It is worth mentioning that the effective masses were not treated as free fitting parameters but were taken from density functional theory (DFT) calculations of the band structure.

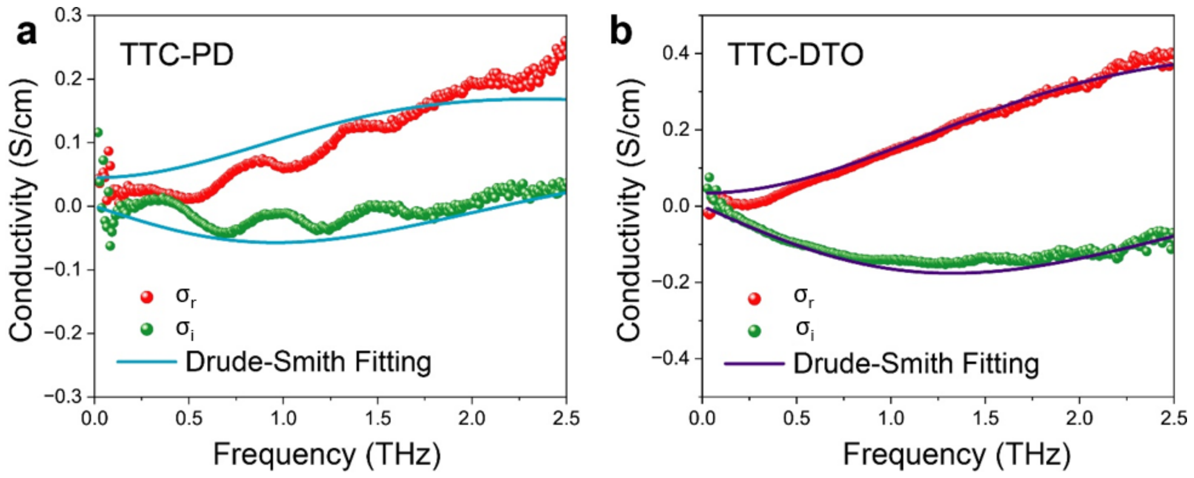

Figure S29: Real and imaginary part of the intrinsic THz conductivity of a) TTC-PD and b) TTC-DTO fitted to the Drude-Smith (DS) Model.

SUPPORTING INFORMATION

Table S5. Scattering time (τ), plasma frequency (ω_p), backscattering parameter (c_1), carrier density (n) and DC mobility (μ_{DC}) of the samples were evaluated from the Drude-Smith (DS) fitting of their respective complex THz conductivity.

**Samples**	***μ****	***τ (ps)***	***$\omega_p/2\pi$ (THz)***	***c_1***	***n (×10^{20} cm^{-3}) (fit)***	***μ_{DC}* (cm²/Vs)**
**TTC-PD**	0.29	0.06315±0.0017	0.12327±0.00214	0.86605±0.01089	0.00355±0.00012	38.3±1.0
**TTC-DTO**	0.62	0.04867±0.0004	0.21485±0.00146	0.95684±0.00296	0.0371±0.0005	13.80±0.11

Table S6: Carrier mobility of TTC-PD and TTC-DTO calculated for carrier masses at different K-path Directions. (μ_{DC}: direct current mobility; m_0: rest mass of electron)

**Material**	**Mass label**	**Effective mass (m_0)**	***n (× 10^{20} cm-3) (fit)***	**μ_{DC} (cm²/Vs)**
**TTC-PD**	***μ****	**0.29**	0.00355±0.00012	**38.3±1.0**
	m_e (in-plane)	0.385	0.00471±0.00017	28.9±0.8
	m_h (in-plane average)	1.26	0.01542±0.00054	8.82±0.24
	Powder average	1.04	0.01273±0.00045	10.7±0.30
	Out of plane	2.53	0.03097± 0.00109	4.39±0.12
**TTC-DTO**	***μ****	**0.62**	0.0371±0.0005	**13.80±0.11**
	m_e (In-plane average)	1.08	0.0647±0.0009	7.93±0.07
	m_h (In-plane average)	2.05	0.1230±0.0017	4.18±0.03
	Powder average	0.58	0.0347±0.0005	14.76±0.12
	Out of plane	0.49	0.0293±0.0004	17.47±0.14

Section S-10: Comparison of charge transport properties TTC-PD and TTC-PD (amor)

Synthesis of TTC-PD (amor):

The amorphous counterpart of TTC-PD, named as TTC-PD (amor) has been synthesized *via* a Schiff base polycondensation reaction between TTC and PD. A 10 mL Schlenk tube was charged with TTC (29.21 μmol, 10 mg) and PD (58.42 μmol, 6.32 mg) with a 1 mL DMF and 0.1 mL (3 M) acetic acid as the catalyst (Scheme S5). The tube was sealed, sonicated, and then degassed by three freeze-pump-thaw cycles. The reaction mixture was heated at 150 ˚C for 3 days. The precipitate formed was collected by filtration and washed with DMF, IPA, water, and acetone. It was further washed in a Soxhlet apparatus with THF for 24 h and then dried under vacuum at 100˚C to obtain TTC-PD (amor) as brown powder in ca. 82 % yield.

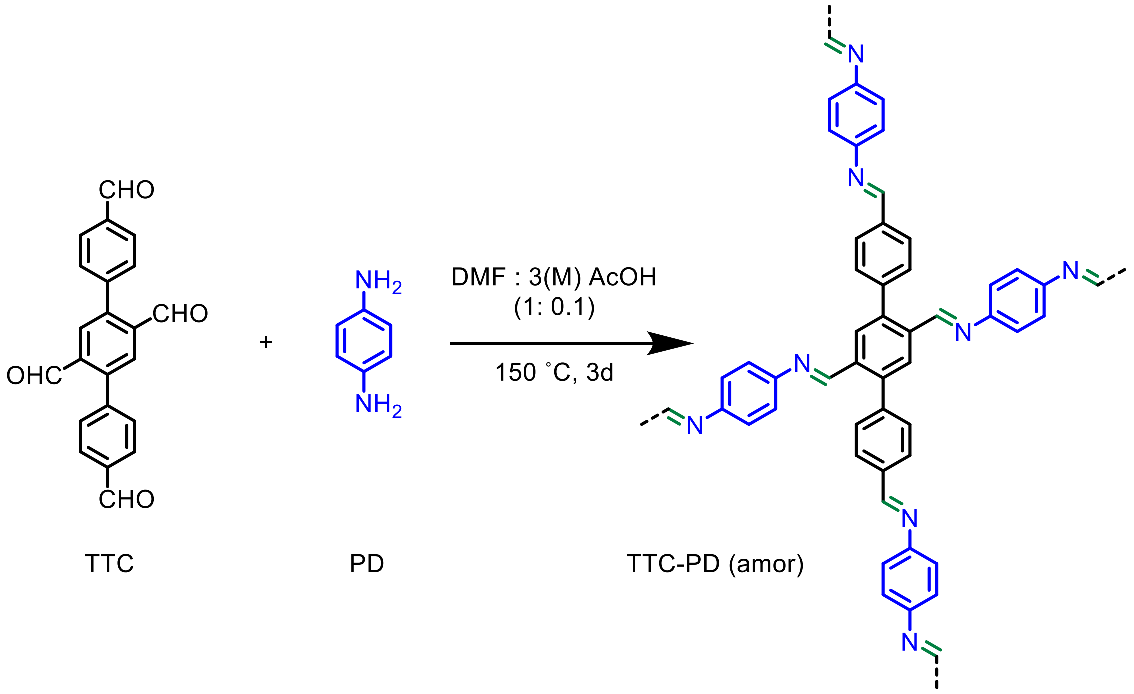

Scheme S5: Schematic representation of the procedure for the synthesis of TTC-PD (amor).

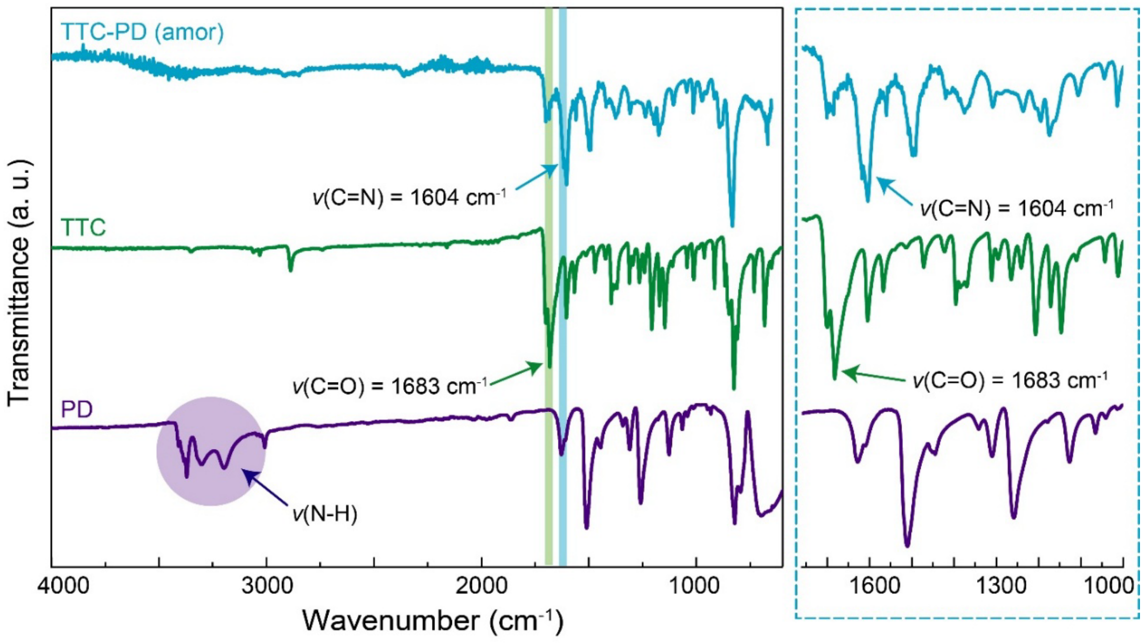

Figure S30: A comparison of the FT-IR spectra of TTC-PD (amor) with its corresponding starting materials, TTC and PD.

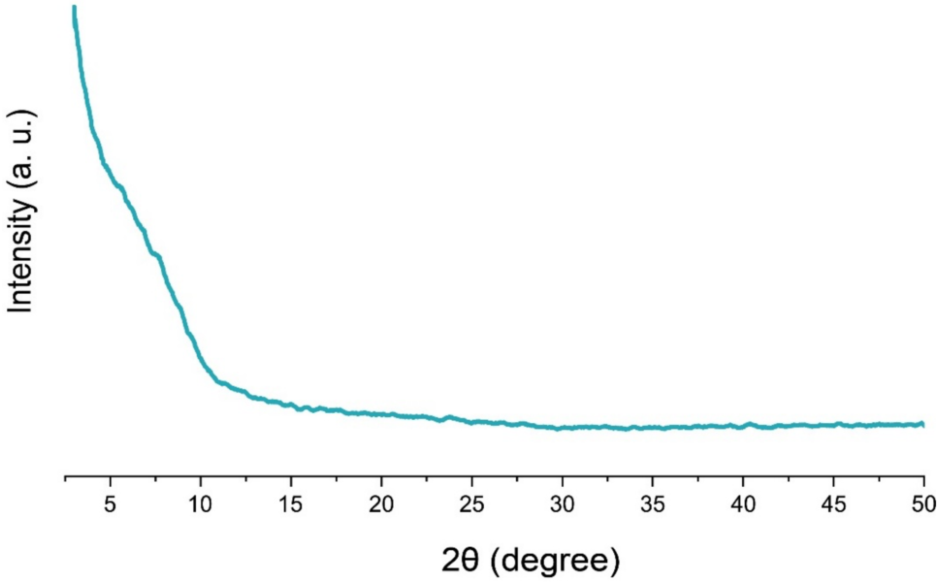

Figure S31: Experimental PXRD pattern of TTC-PD (amor), revealing its amorphous nature.

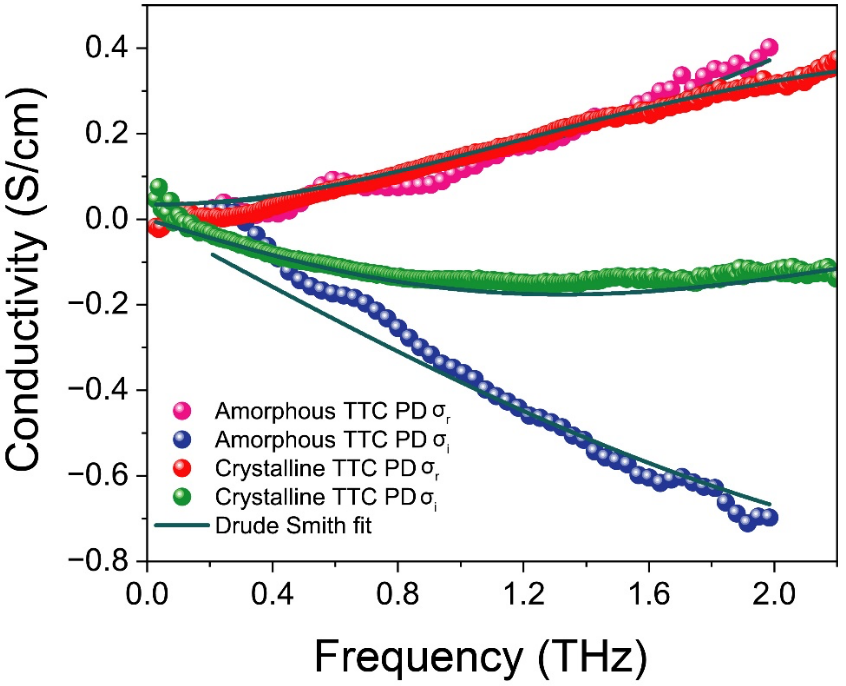

Figure S32: Comparison of the real and imaginary parts of the intrinsic THz conductivity of TTC-PD and TTC-PD (amor) fitted to the DS Model.

Table S7. Comparison of Scattering time (τ), and backscattering parameter (c_1), of TTC-PD and TTC-PD (amor), evaluated from the DS fitting of their respective complex THz conductivity.

Samples	τ (ps)	c_1
**TTC-PD**	0.06315±0.0017	0.86605±0.01089
**TTC-PD (amor)**	0.01902 ± 0.001	-0.9891 ± 0.00232

Section S-11: Temperature-dependent time-domain THz spectroscopy

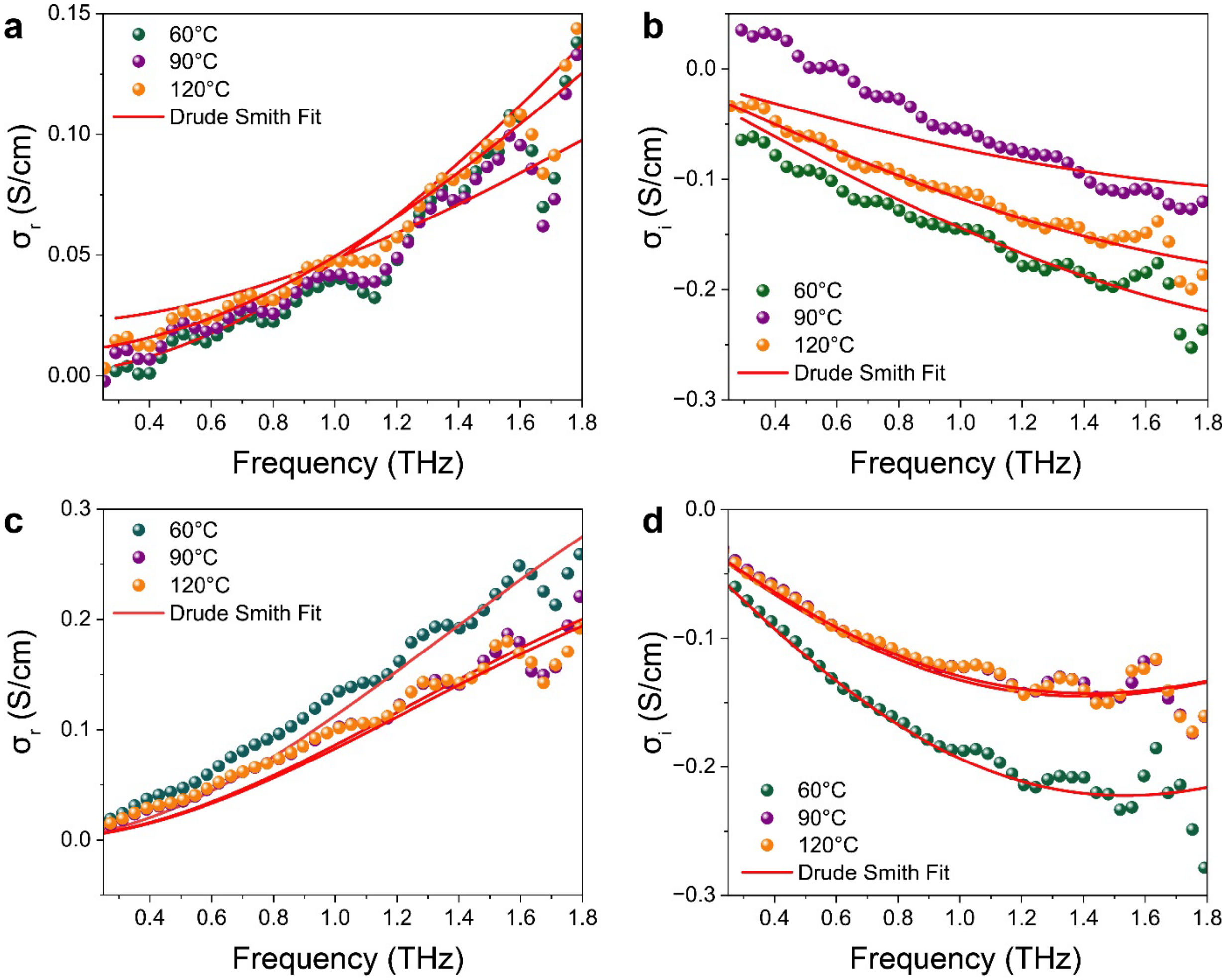

Figure S33: Temperature-dependent real and imaginary conductivity spectra with DS fits for (a, b) TTC-PD and (c, d) TTC-DTO at 60, 90, and 120 °C.

Table S8: Scattering time (τ), plasma frequency (ω_p), and backscattering parameter (c_1) of the TTC-PD evaluated from the DS fitting of their respective complex THz conductivity at different temperatures.

T (°C)	$\omega_p/2\pi$ (THz)	c_1	τ (ps)
**RT**	0.12327 ± 0.00214	-0.86± 0.01089	0.06315 ± 0.0017
**60**	0.22744 ± 0.00327	-1 ± 0.00851	0.03767 ± 0.0006
**90**	0.22988 ± 0.00421	-0.95607 ± 0.00775	0.02735 ± 0.0005567
**120**	0.29101 ± 0.00879	-0.9885 ± 0.00276	0.02649 ± 0.0008507

Table S9: Carrier density (n) and DC mobility (μ_{DC}) of the TTC-PD evaluated from the DS fitting of their respective complex THz conductivity at different temperatures, calculated for carrier masses at different K-path Directions.

T (°C)	m^*/m_0	n ($\times 10^{20}$ cm^{-3})	μ_{DC} (cm^2/Vs)
RT	0.29	0.00355 ± 0.00012	38.3 ± 1.0
RT	0.385	0.00471 ± 0.00017	28.9 ± 0.8
RT	1.04	0.01273 ± 0.00045	10.7 ± 0.3

RT	1.26	0.01542 ± 0.00054	8.82 ± 0.24
RT	2.53	0.03097 ± 0.00109	4.39 ± 0.12
60	0.29	0.0180 ± 0.0012	22.9 ± 0.4
60	0.385	0.0239 ± 0.0016	17.2 ± 0.3
60	1.04	0.0646 ± 0.0043	6.37 ± 0.10
60	1.26	0.0782 ± 0.0052	5.26 ± 0.08
60	2.53	0.157 ± 0.010	2.62 ± 0.04
90	0.29	0.0201 ± 0.0013	16.6 ± 0.3
90	0.385	0.0267 ± 0.0018	12.5 ± 0.3
90	1.04	0.0721 ± 0.0048	4.62 ± 0.10
90	1.26	0.0873 ± 0.0058	3.81 ± 0.08
90	2.53	0.175 ± 0.012	1.90 ± 0.04
120	0.29	0.0333 ± 0.0022	15.8 ± 0.5
120	0.385	0.0442 ± 0.0029	11.9 ± 0.4
120	1.04	0.119 ± 0.008	4.40 ± 0.15
120	1.26	0.144 ± 0.010	3.63 ± 0.12
120	2.53	0.290 ± 0.019	1.81 ± 0.06

Table S10: Scattering time (τ), plasma frequency (ω_p), and backscattering parameter (c_1) of the TTC-DTO evaluated from the DS fitting of their respective complex THz conductivity at different temperatures.

**T (°C)**	**$\omega_p/2\pi$ (THz)**	**c_1**	**τ (ps)**
RT	0.21485 ± 0.00146	-0.95684 ± 0.00296	0.04867 ± 0.0004
60	0.26513 ± 0.00542	-0.97629 ± 0.00389	0.03891 ± 0.0009
90	0.22676 ± 0.00546	-0.93919 ± 0.00377	0.03738 ± 0.001
120	0.224 ± 0.00126	-0.95597 ± 0.00306	0.03715 ± 0.000219

Table S11: Carrier density (n) and DC mobility (μ_{DC}) of the TTC-DTO evaluated from the DS fitting of their respective complex THz conductivity at different temperatures, calculated for carrier masses at different K-path Directions.

**T (°C)**	**m^*/m_0**	**n (×10^{20} cm^{-3})**	**μ_{DC} (cm^2/Vs)**
RT	0.62	0.0371 ± 0.0005	13.80 ± 0.11
RT	1.08	0.0647 ± 0.0009	7.93 ± 0.07
RT	2.05	0.1230 ± 0.0017	4.18 ± 0.03
RT	0.58	0.0347 ± 0.0005	14.76 ± 0.12
RT	0.49	0.0293 ± 0.0004	17.47 ± 0.14
60	0.62	0.0515 ± 0.0020	11.04 ± 0.27
60	1.08	0.0897 ± 0.0035	6.34 ± 0.16
60	2.05	0.170 ± 0.0066	3.34 ± 0.08
60	0.58	0.0481 ± 0.0019	11.80 ± 0.29
60	0.49	0.0407 ± 0.0016	13.97 ± 0.34
90	0.62	0.0378 ± 0.0017	11.37 ± 0.30

90	1.08	0.0659 ± 0.0030	6.53 ± 0.17
90	2.05	0.125 ± 0.0056	3.44 ± 0.09
90	0.58	0.0353 ± 0.0016	12.16 ± 0.32
90	0.49	0.0299 ± 0.0013	14.39 ± 0.38
120	0.62	0.0370 ± 0.0004	11.40 ± 0.07
120	1.08	0.0645 ± 0.0007	6.55 ± 0.04
120	2.05	0.122 ± 0.0013	3.45 ± 0.02
120	0.58	0.0346 ± 0.0004	12.18 ± 0.08
120	0.49	0.0292 ± 0.0003	14.42 ± 0.09

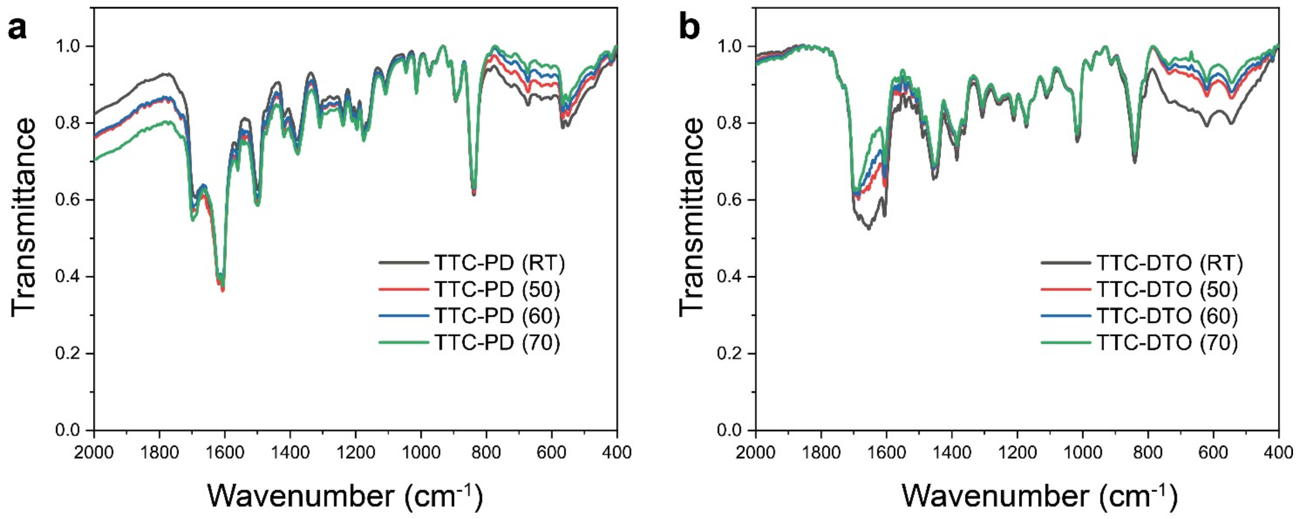

Figure S34: Temperature-dependent FTIR spectra of a) TTC-PD and b) TTC-DTO.

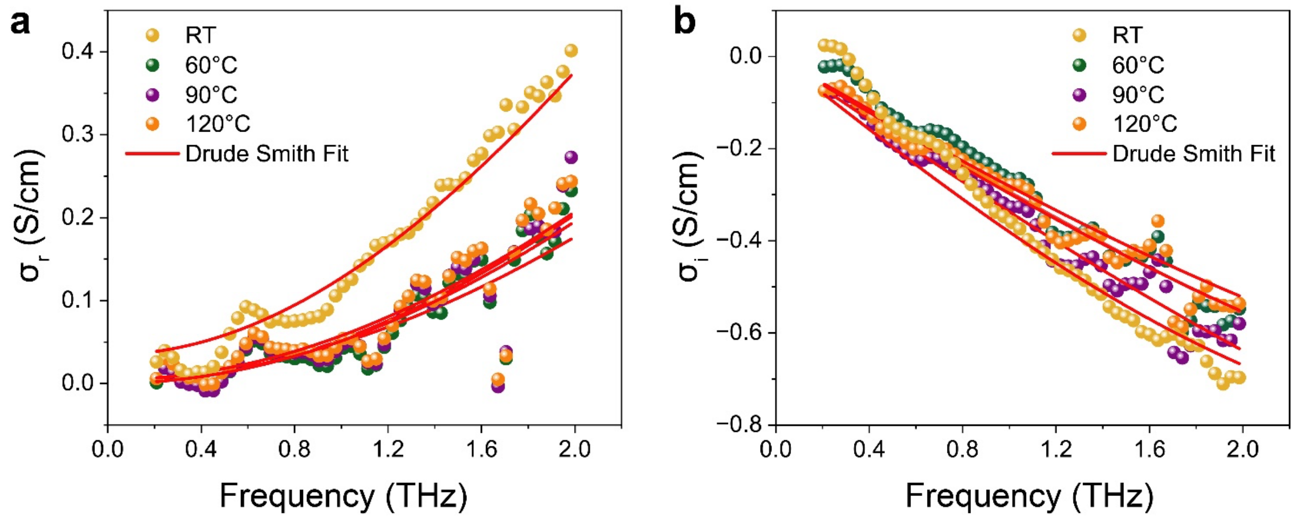

Figure S35: a) Real part and b) imaginary part of the temperature-dependent conductivity spectra of TTC-PD(amor) with DS fit at RT, 60, 90, and 120 °C.

Table S12: plasma frequency (ω_p), scattering time (τ), and backscattering parameter (c_1) of the TTC-PD (amor) evaluated from the DS fitting of their respective complex THz conductivity at various temperatures.

T (°C)	$\omega_p/2\pi$ (THz)	c_1	τ (ps)
RT	0.71465 ± 0.03628	-0.9891 ± 0.00232	0.01902 ± 0.001
60	0.9717 ± 0.09449	-0.99892 ± 0.02	0.01201 ± 0.00118
90	1.00785 ± 0.07013	-1 ± 0.0174	0.0124 ± 0.0009
120	1.09853 ± 0.00731	-0.9948 ± 0.0109	0.01069 ± 0.00007

Table S13: Carrier density (*n*) and DC mobility (μ_{DC}) of the TTC-PD (amor) evaluated from the DS fitting of their respective complex THz conductivity, calculated for carrier masses at different K-path Directions.

T (°C)	m^*/m_0	n ($\times 10^{20}$ cm^{-3})	μ_{DC} (cm^2/Vs)
RT	0.29	0.179 ± 0.018	11.55 ± 0.61
RT	0.385	0.238 ± 0.024	8.69 ± 0.46
RT	1.04	0.643 ± 0.065	3.22 ± 0.17
RT	1.26	0.779 ± 0.079	2.66 ± 0.14
RT	2.53	1.564 ± 0.158	1.32 ± 0.07
60	0.29	0.331 ± 0.064	7.29 ± 0.72
60	0.385	0.440 ± 0.085	5.49 ± 0.54
60	1.04	1.188 ± 0.230	2.03 ± 0.20
60	1.26	1.439 ± 0.279	1.68 ± 0.17
60	2.53	2.891 ± 0.560	0.83 ± 0.08
90	0.29	0.356 ± 0.050	7.52 ± 0.55
90	0.385	0.473 ± 0.066	5.66 ± 0.41
90	1.04	1.277 ± 0.179	2.09 ± 0.15
90	1.26	1.546 ± 0.217	1.73 ± 0.13
90	2.53	3.106 ± 0.436	0.86 ± 0.06
120	0.29	0.423 ± 0.006	6.49 ± 0.04
120	0.385	0.562 ± 0.008	4.88 ± 0.03
120	1.04	1.518 ± 0.021	1.81 ± 0.01
120	1.26	1.838 ± 0.026	1.49 ± 0.01
120	2.53	3.692 ± 0.052	0.74 ± 0.005

Section S-12: Theoretical studies

Computational method adopted:

To understand the presence of different vibrational features in molecular analogues (Bn-PD and Bn-DTO) that are not present in fragment material, we took the help of the DFT method. All geometry optimizations and frequency calculations were carried out using the B3LYP hybrid functional in combination with the 6-311+G(d,p) basis set.[58,59,66] For computational efficiency and clarity of interpretation, we have taken a small monomer unit for starting material Bn-PD and Bn-DTO, also for TTC-PD and TTC-DTO, for the optimization and frequency calculation.

Table S14: Optimized geometry co-ordinates of TTC-PD, TTC-DTO, Bn-PD, and Bn-DTO

TTC-PD imaginary frequency = 0, E= -881.172421 a.u				TTC-DTO imaginary frequency = 0, E= -2888.419713 a.u			
C	-0.715032000	-1.183650000	-0.012333000	C	-5.020579000	-4.333973000	-0.506945000
C	0.683918000	-1.238820000	-0.039317000	C	-3.991909000	-4.477877000	0.376781000
C	1.420392000	-0.028455000	-0.050730000	S	-5.138629000	-2.684164000	-1.007439000
C	-2.903377000	-0.026451000	0.107349000	N	-3.289288000	-3.338009000	0.664106000
C	3.599059000	-0.636182000	-1.162765000	S	0.287149000	3.167682000	1.200230000
C	4.984225000	-0.548725000	-1.251390000	C	-1.017356000	2.775187000	0.080041000
C	5.718158000	0.192900000	-0.317090000	N	-1.438411000	3.803757000	-0.605996000
C	5.026810000	0.854620000	0.709512000	C	-1.664395000	1.448364000	0.031769000
C	3.645749000	0.769972000	0.794023000	C	-0.972811000	0.218580000	0.080750000
C	-1.351020000	2.539791000	-0.064446000	C	-1.722934000	-0.958079000	0.109700000
C	-7.174972000	-0.273475000	0.424592000	C	-3.122976000	-0.970135000	0.068864000
N	-0.710442000	3.634903000	0.091517000	C	-3.814184000	0.259570000	0.016810000
N	7.912622000	0.890670000	0.398087000	C	-3.063585000	1.436152000	-0.010153000
H	-1.253702000	-2.122334000	0.028336000	C	-3.765826000	-2.299272000	0.030035000
H	3.048702000	-1.198137000	-1.908677000	C	0.510429000	0.102655000	0.027114000
H	5.502739000	-1.056529000	-2.058513000	C	-5.300170000	0.378041000	0.058185000
H	5.593189000	1.427788000	1.433265000	C	1.240970000	0.672528000	-1.024792000
H	3.124622000	1.280624000	1.596078000	C	2.617765000	0.519522000	-1.100860000
H	2.420530000	-2.539751000	0.258791000	C	3.314634000	-0.206022000	-0.123284000
H	-7.600867000	0.234429000	1.300978000	C	2.585453000	-0.783692000	0.927765000
C	0.717072000	1.174697000	-0.016810000	C	1.207219000	-0.634654000	0.994835000
C	-0.681921000	1.230013000	0.007980000	C	-5.988058000	1.049274000	-0.960366000
C	-1.417707000	0.019768000	0.019539000	C	-7.372347000	1.199929000	-0.902558000
C	2.905670000	0.018449000	-0.135871000	C	-8.087100000	0.692339000	0.180427000
C	-3.588771000	0.589923000	1.162671000	C	-7.409840000	0.033589000	1.206508000
C	-4.973492000	0.501363000	1.257120000	C	-6.028581000	-0.124005000	1.145340000
C	-5.714667000	-0.202139000	0.299113000	C	6.766330000	-1.033074000	0.417951000
C	-5.030944000	-0.825250000	-0.756126000	C	7.160008000	-0.351882000	-0.720391000
C	-3.649927000	-0.740676000	-0.845595000	S	8.177644000	-1.718079000	1.176834000
C	1.351561000	-2.548651000	0.026683000	N	8.482834000	-0.334875000	-1.029283000

C	7.179026000	0.261712000	-0.436002000
N	0.720214000	-3.643064000	-0.170369000
N	-7.914973000	-0.866258000	-0.429875000
H	1.254634000	2.113743000	-0.057981000
H	-3.030722000	1.119409000	1.926762000
H	-5.486419000	0.976054000	2.087763000
H	-5.602999000	-1.369410000	-1.497573000
H	-3.134144000	-1.222211000	-1.668798000
H	-2.419813000	2.527913000	-0.297949000
H	7.611296000	-0.281111000	-1.287908000
C	-1.381120000	4.868845000	0.024885000
C	-0.684081000	5.956265000	-0.522349000
C	-2.677694000	5.074882000	0.523943000
C	-1.287277000	7.205444000	-0.615984000
C	-3.268582000	6.332774000	0.443950000
C	-2.582166000	7.400356000	-0.133523000
H	0.326752000	5.795579000	-0.878088000
H	-3.208774000	4.258401000	0.999484000
H	-0.741760000	8.033003000	-1.055628000
H	-4.266284000	6.481573000	0.842168000
H	-3.045789000	8.378276000	-0.192928000
C	9.302239000	0.970812000	0.200745000
C	9.886457000	1.180948000	-1.058579000
C	10.130805000	0.892446000	1.329795000
C	11.270215000	1.276170000	-1.183014000
C	11.512410000	0.969852000	1.195461000
C	12.089454000	1.161758000	-0.060973000
H	9.254066000	1.304295000	-1.930423000
H	9.670113000	0.759813000	2.301589000
H	11.708725000	1.450569000	-2.159476000
H	12.141781000	0.891996000	2.075044000
H	13.165963000	1.237928000	-0.161613000
C	-9.303506000	-0.952125000	-0.227312000
C	-9.880089000	-1.214424000	1.025706000
C	-10.138906000	-0.824203000	-1.346729000
C	-11.263247000	-1.312230000	1.154843000
C	-11.519795000	-0.904524000	-1.206897000
C	-12.089268000	-1.148580000	0.043938000
C	9.133789000	-1.009089000	-0.124941000
S	5.752828000	0.333845000	-1.480477000
C	4.768918000	-0.378758000	-0.168279000
N	5.449448000	-1.051050000	0.727103000
H	-1.208454000	-1.910261000	0.114125000
H	-3.580118000	2.387378000	-0.016296000
H	0.723009000	1.231465000	-1.795423000
H	3.149046000	0.966759000	-1.933778000
H	3.115201000	-1.345371000	1.686270000
H	0.661528000	-1.084144000	1.816904000
H	-5.435746000	1.443778000	-1.806154000
H	-7.890931000	1.714353000	-1.703887000
H	-7.957781000	-0.354246000	2.058008000
H	-5.506628000	-0.632117000	1.948498000
C	-0.740675000	4.942106000	-0.297268000
C	0.226464000	4.806526000	0.654410000
H	0.896940000	5.554259000	1.048492000
H	-0.980300000	5.864031000	-0.809781000
H	10.206045000	-1.149671000	-0.136573000
H	-9.163603000	0.812147000	0.227320000
H	-3.708614000	-5.407365000	0.852084000
H	-5.707443000	-5.081389000	-0.872390000

H	-9.242414000	-1.376272000	1.887351000
H	-9.684078000	-0.651215000	-2.314925000
H	-11.695960000	-1.527368000	2.125746000
H	-12.154547000	-0.788099000	-2.078305000
H	-13.165279000	-1.226954000	0.148131000
C	1.376342000	-4.875479000	-0.003943000
C	2.308586000	-5.120790000	1.017344000
C	1.032945000	-5.919529000	-0.875687000
C	2.904406000	-6.373512000	1.136804000
C	1.645329000	-7.162453000	-0.761667000
C	2.584197000	-7.395787000	0.244450000
H	2.538973000	-4.341355000	1.734640000
H	0.290295000	-5.730055000	-1.641743000
H	3.614180000	-6.554146000	1.936609000
H	1.381191000	-7.956051000	-1.451762000
H	3.049060000	-8.370057000	0.342137000

Bn-PD imaginary frequency = 0, E= -881.172421 a.u

C	5.606809000	1.089762000	-0.207640000
C	5.076809000	-0.183900000	0.054411000
C	5.954237000	-1.257482000	0.259364000
H	7.383799000	2.263643000	-0.462469000
H	8.921648000	0.351781000	-0.096998000
H	4.922643000	1.914322000	-0.365871000
H	3.327815000	-1.456193000	0.318620000
C	7.332890000	-1.067534000	0.205633000
C	7.848870000	0.200339000	-0.054145000
C	6.981369000	1.277350000	-0.259882000
H	5.551239000	-2.244799000	0.462302000
C	3.629467000	-0.418853000	0.118143000
N	2.767490000	0.512235000	-0.028315000
H	8.001786000	-1.905463000	0.365852000
C	1.394248000	0.221666000	-0.021614000
C	0.528334000	1.156118000	0.564903000
C	0.840672000	-0.932457000	-0.602633000
C	-0.840753000	0.932947000	0.603114000
C	-0.528400000	-1.155589000	-0.564460000
C	-1.394304000	-0.221185000	0.022189000
H	0.955484000	2.046804000	1.010969000

Bn-DTO imaginary frequency = 0, E= -1521.625626 a.u

H	7.565987000	0.251676000	-0.004943000
C	6.483850000	0.188543000	-0.002361000
C	5.854937000	-1.051919000	0.093496000
C	4.466569000	-1.134333000	0.097645000
C	3.682631000	0.025106000	0.003435000
C	4.324947000	1.271107000	-0.091896000
C	5.712125000	1.347683000	-0.094055000
C	0.150720000	0.675196000	-0.004825000
C	-0.150724000	-0.675204000	-0.004774000
S	-1.336117000	1.581549000	-0.002187000
N	-1.452076000	-1.038756000	-0.000610000
C	-2.217681000	0.025364000	0.003554000
S	1.336118000	-1.581557000	-0.002075000
C	2.217681000	-0.025361000	0.003527000
N	1.452072000	1.038753000	-0.000703000
H	6.445724000	-1.957707000	0.167415000
H	3.994945000	-2.107470000	0.178962000
H	3.720286000	2.165869000	-0.163931000
H	6.194299000	2.315843000	-0.168956000
C	-3.682630000	-0.025105000	0.003451000
C	-4.466566000	1.134329000	0.097740000

H	1.485462000	-1.644272000	-1.105258000	C	-4.324947000	-1.271098000	-0.091970000
H	-1.485526000	1.644865000	1.105621000	C	-5.854934000	1.051918000	0.093580000
H	-0.955603000	-2.046234000	-1.010563000	C	-5.712126000	-1.347671000	-0.094138000
N	-2.767500000	-0.511829000	0.028825000	C	-6.483850000	-0.188536000	-0.002365000
C	-3.629490000	0.419258000	-0.117421000	H	-3.994940000	2.107458000	0.179141000
H	-3.327931000	1.456710000	-0.317448000	H	-3.720289000	-2.165856000	-0.164068000
C	-5.076809000	0.183985000	-0.054102000	H	-6.445720000	1.957702000	0.167565000
C	-5.954477000	1.257080000	-0.260532000	H	-6.194301000	-2.315826000	-0.169111000
C	-5.606519000	-1.089641000	0.208681000	H	-7.565987000	-0.251667000	-0.004954000
C	-7.333098000	1.066686000	-0.207481000				
C	-6.981045000	-1.277690000	0.260184000				
C	-7.848795000	-0.201160000	0.052993000				
H	-5.551702000	2.244354000	-0.464119000				
H	-4.922155000	-1.913823000	0.368012000				
H	-8.002198000	1.904224000	-0.368892000				
H	-7.383232000	-2.263982000	0.463276000				
H	-8.921551000	-0.352963000	0.095169000				

Section S-13: References

[60] Z. Jamain, M. Khairuddean, *J. Phys. Conf. Ser.* **2021**, *1882*, 012120.

[61] B. P. Biswal, D. Becker, N. Chandrasekhar, J. S. Seenath, S. Paasch, S. Machill, F. Hennersdorf, E. Brunner, J. J. Weigand, R. Berger, X. Feng, *Chem. Eur. J.* **2018**, *24*, 10868-10875.

[62] G. Kresse, J. Furthmüller, *Phys. Rev. B* **1996**, *54*, 11169-11186.

[63] G. Kresse, J. Furthmüller, *Comput. Mater. Sci.* **1996**, *6*, 15-50.

[64] J. P. Perdew, K. Burke, M. Ernzerhof, *Phys. Rev. Lett.* **1996**, *77*, 3865–3868.

[65] V. Wang, N. Xu, J.-C. Liu, G. Tang, W.-T. Geng, *Comput. Phys. Commun.* **2021**, *267*, 108033.

[66] S. Jena, K. D. Tulsiyan, R. R. Sahoo, S. Rout, A. K. Sahu, H. S. Biswal, *Chem. Sci.* **2023**, *14*, 14200-14210.
